# Transformation of Stilbene Glucosides From *Reynoutria multiflora* During Processing

**DOI:** 10.3389/fphar.2022.757490

**Published:** 2022-04-25

**Authors:** Junqi Bai, Wanting Chen, Juan Huang, He Su, Danchun Zhang, Wen Xu, Jing Zhang, Zhihai Huang, Xiaohui Qiu

**Affiliations:** ^1^ Guangdong Provincial Hospital of Traditional Chinese Medicine, The Second Clinical Medical College of Guangzhou University of Chinese Medicine, Guangzhou, China; ^2^ Guangzhou Key Laboratory of Chirality Research on Active Components of Traditional Chinese Medicine, Guangzhou, China; ^3^ Guangdong Provincial Key Laboratory of Clinical Research on Traditional Chinese Medicine Syndrome, Guangzhou, China

**Keywords:** *Reynoutria multiflora*, stilbene glycosides, processed, UHPLC-Q-Exactive plus orbitrap MS/MS, structural and content changes

## Abstract

The root of *Reynoutria multiflora* Thunb. Moldenke (RM, syn.: *Polygonum multiflorum* Thunb.) has been widely used in TCM clinical practice for centuries. The raw *R. multiflora* (RRM) should be processed before use, in order to reduce toxicity and increase efficiency. However, the content of trans-2, 3, 5, 4′-tetrahydroxystilbene-2-O-β-D-glucopyranoside (trans-THSG), which is considered to be the main medicinal ingredient, decreases in this process. In order to understand the changes of stilbene glycosides raw *R. multiflora* (RRM) and processed *R. multiflora* (PRM), a simple and effective method was developed by ultra high performance liquid chromatography tandem quadrupole/electrostatic field orbitrap high-resolution mass spectrometry (UHPLC-Q-Exactive plus orbitrap MS/MS). The content and quantity of stilbene glycosideshave undergone tremendous changes during the process. Seven parent nucleus of stilbene glycosides and 55 substituents, including 5-HMF and a series of derivatives, were identified in PM. 146 stilbene glycosides were detected in RRM, The number of detected compounds increased from 198 to 219 as the processing time increased from 4 to 32 h. Among the detected compounds, 102 stilbene glycosides may be potential new compounds. And the changing trend of the compounds can be summarized in 3 forms: gradually increased, gradually decreased, first increased and then decreased or decreased first. The content of trans-THSG was indeed decreased during processing, as it was converted into a series of derivatives through the esterification reaction with small molecular compounds. The clarification of secondary metabolite group can provide a basis for the follow-up study on the mechanism of pharmacodynamics and toxicity of PM, and for screening of relevant quality markers.

## 1 Introduction

Traditional Chinese medicine processing is a unique pharmaceutical technology derived from the theory of traditional Chinese medicine. It has played a prominent role in the clinical practice of traditional Chinese medicine for thousands of years, ensuring the safety and effectiveness of treatment. After processing with different temperatures, durations, solvents or excipients, the components of traditional Chinese medicine have undergone different changes. Ingredients will be dissolved, decomposed or transformed into new components, resulting in increasing or decreasing of the compounds. All these changes are closely related to the property and efficacy of traditional Chinese medicine. Therefore, it is of great significance to study the changes of chemical components before and after processing of traditional Chinese medicine.

The root of *Reynoutria multiflora* Thunb. (*Polygonum multiflorum* Thunb.), well known as He-shou-wu in China, has been widely used in TCM clinical practice for centuries (Li et al., 2017). Lots of research have shown that RRM and its processed products have different pharmacological effects. RRM has the effect of detoxification, carbuncle elimination, relaxing bowel. And PRM shows the effect of tonifying liver and kidney, tonic medicines and hair-blacking (Cheung et al., 2014; Lin et al., 2015; Chinese Pharmacopoeias Commission, 2020). RRM is commonly processed by steaming with black bean or water, which has been officially documented in the Chinese pharmacopoeia. However, the processing time was not specified in the processing specification. Therefore, the processing time of PRM on the market varies greatly, ranging from 2 to 18 h (Lin et al., 2018). But in our previous studies, we have screened out that the best effect of PRM was processing for 24–32 h (Qiu et al., 2008). The quality of PRM is inhomogeneous in the market, the main reason for this phenomenon is that the processing mechanism of PRM is not clear. The increased reports of hepatotoxicity of RRM in recent years (Dong et al., 2014; Lei et al., 2015; Zhang et al., 2019) may also be related to incomplete processing.

Previous research indicated that the main chemical components of RM were secondary metabolites, including stilbene glycosides, anthraquinone and polyphenols were the most representative (Choi et al., 2007; Lin et al., 2015; Sun et al., 2015). The fragmentation pathways of typical constituents and chemical profiles of RM have been studied by an on-line UHPLC-ESI-linear ion trap-Orbitrap hybrid mass spectrometry method (Xu et al., 2012; Qiu et al., 2013). The secondary metabolites were quantitatively analyzed by HPLC/LC-MS/MS to study the chemical components before and after processing of *R. multiflora*, which showed that the content of some chemical substances was changed by processing. In our previous study, the contents of 5-HMF, THSG, emodin and physcion are changed during the processing (Chen et al., 2012). The content of THSG, a compound that possess anti-oxidative, anti-aging, anti-tumor, anti-inflammatory and liver protective activities (Lv et al., 2007; Shao et al., 2012; Lin et al., 2015; Yang et al., 2020), was decreased (Qiu et al., 2006; Fu, 2011). However, there is no research report on the secondary metabolite group produced by stilbene glycosides in the process, and the clarification of secondary metabolite group can provide a basis for the follow-up study on the mechanism of pharmacodynamics and toxicity of PM, and for screening of relevant quality markers.

In this study, a simple and rapid method for the determination of RRM and PRM by UHPLC-Q-Exactive plus orbitrap MS/MS was established, and the qualitative analysis of RRM and PRM were carried out *in vitro* to obtain a clear chemical map. The fragment ions at *m/z* 405.1087 and 243.0656 were selected as characteristic fragments, the secondary metabolites in RRM and processed PRM samples prepared with different durations were characterized and identified, then, the changes of stilbene glycosides during processing were further analyzed.

## 2 Materials and Methods

### 2.1 Materials

RRM and PRMs that had been processed for 4, 8, 12, 18, 24 and 32 h were provided by Shanghai Dehua Traditional Chinese Medicine CO., Ltd., and the corresponding batch numbers were HSW2018051101-S, HSW2018051101-4H, HSW2018051101-8H, HSW2018051101-12H, HSW2018051101-18H, HSW2018051101-24H, and HSW2018051101-32H. The samples were authenticated by Professor Zhihai Huang, and voucher specimens were deposited in the Materials Medica Preparation Lab of the Second Affiliated Hospital of the Guangzhou University of Chinese Medicine.

Trans-2, 3, 5, 4′-tetrahydroxystilbene-2-O-β-D-glucopyranoside (THSG), cis-THSG and polydatin were purchased from yuanye Bio-Technology Co., Ltd. Acetonitrile (No. H08J11E115101, P27A11P107214, T15A10F85743, purity ≥98%, Shanghai, China). acetonitrile and methanol (HPLC grade), were supplied by E. Merck (Darmstadt, Germany), formic acid (HPLC grade) was purchased from fisher (United States), ultra-pure water was prepared by a Mili-Q water purification system (Millipore, MA, United States).

### 2.2 Sample Processing Method

All the samples were prepared using following method: 1 g sample powder was ultrasonicated for 30 min with 25 ml of 70% ethanol, followed by filtration and then evaporated the filtrate. 5 ml of ultrapure water were added to dissolve the residue and then extracted twice with 15 ml of ethyl acetate. The resulting mixture was combined with an ethyl acetate solution and evaporated over a water bath; after that, 1 ml of methanol was added to dissolve the residue and centrifugation (15,000 rpm, 4°C) for 10 min by a 1.5 ml centrifuge tube. Finally, the supernatant of the treatment samples was injected into the UPLC-Q-Exactive plus orbitrap MS/MS system.

### 2.3 UHPLC-Q-Exactive Plus Orbitrap MS/MS Analysis

#### 2.3.1 Liquid Chromatography

All the samples were analysed using an Ultimate 3000 UPLC system (Dionex, United States) that was controlled with Thermo Xcalibur software (Thermo Fisher Scientific, United States). The samples were separated using a Kinetex UPLC C18 column (100*2.1 mm, 1.7 µm) (Phenomenex, United States). The mobile phase consisted of solvent A (0.1% formic acid) and solvent B (acetonitrile). A gradient elution was applied using the following optimized gradient program: 8-8% B at 0–3 min, 8–28% B at 3–25 min, 28–40% B at 25–26 min, 40–50% B at 26–28 min, 50–70% B at 28–30 min, 70–90% B at 30–32 min, and 90–90% B at 32–35 min. The flow rate was kept at 0.4 ml/min, the sample injection volume was 1 μL, and the column temperature was maintained at 25°C.

### 2.4 Mass Spectrometry

Mass spectrometry was performed on a Q-Exactive Plus™ quadrupole-Orbitrap mass spectrometer (Thermo Fisher Scientific, United States) in negative ion mode. The scan mass range was set at *m/z* 100–1,200. The parameter settings were as follows: a full scan and fragment spectral resolution of 70,000 FWHM and 17, 50 FWHM, respectively; capillary temperature was 350°C; auxiliary gas heater temperature was 350°C; spray voltage was −3.2 KV; sheath gas flow rate was 40 Arb; auxiliary gas flow rate was 15 Arb; and S-lens RF level was set at 50. The acquisition mode of stepped NCE (normalized collision energy) was using with settings of 30, 50, and 70 eV. The accumulated resultant fragment ions were injected into the Orbitrap mass analyzer for single-scan detection.

Considering the possible elemental composition of the RM components, the types and quantities of expected atoms were set as follows: carbon ≤50, hydrogen ≤200, oxygen ≤20, nitrogen ≤3. The accuracy error threshold was fixed at 5 ppm.

## 3 Results and Discussion

### 3.1 Base Peak Chromatograms

**FIGURE 1:**
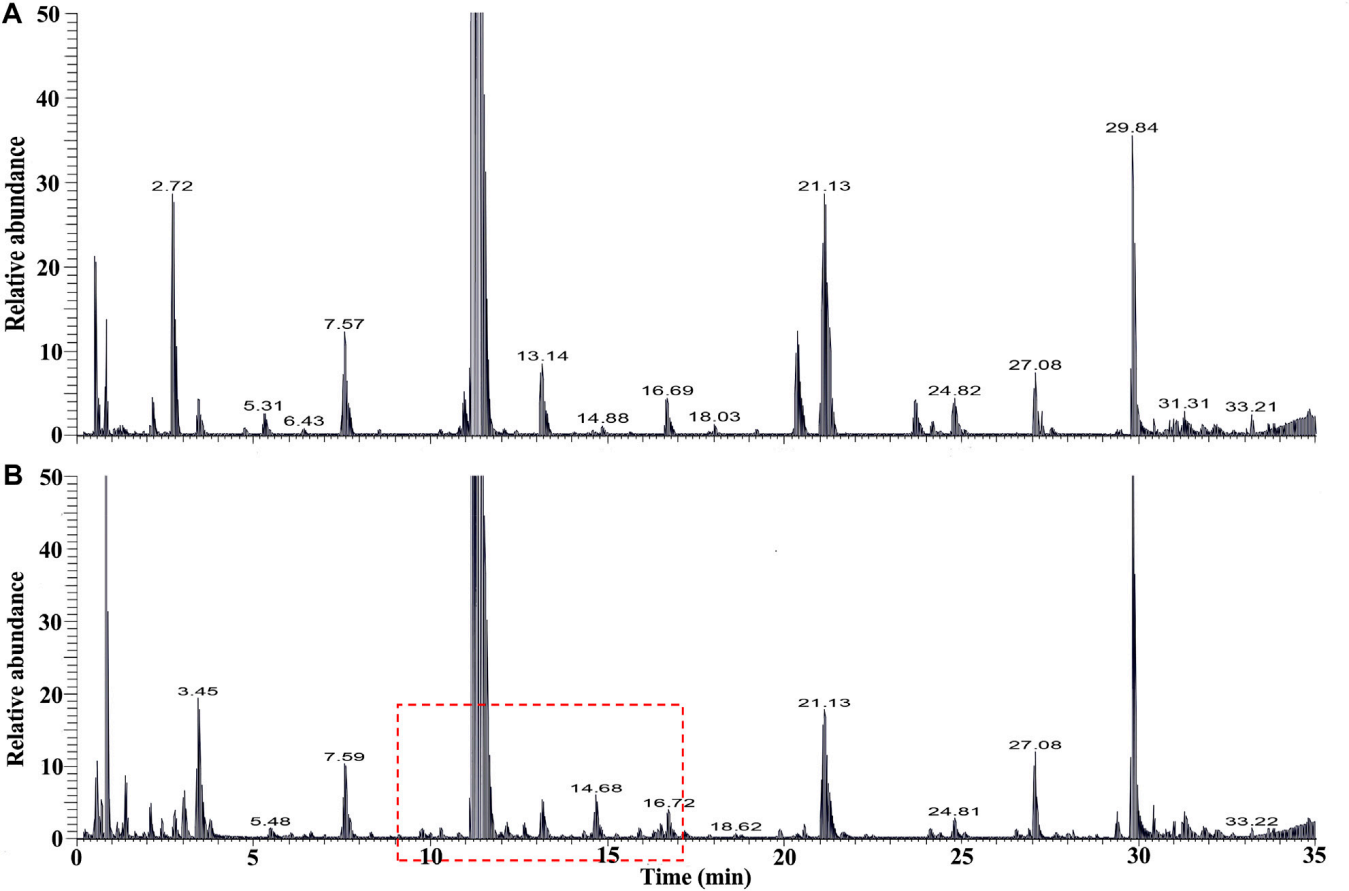
The Base peak intensity chromatograms of samples of *raw Reynoutria multiflora* Thunb. [RRM, **(A)**] and processed *Reynoutria multiflora* Thunb. [PRM for 24h, **(B)**] derived from UHPLC-Q-Exactive plus orbitrap MS/MS.

**FIGURE 2:**
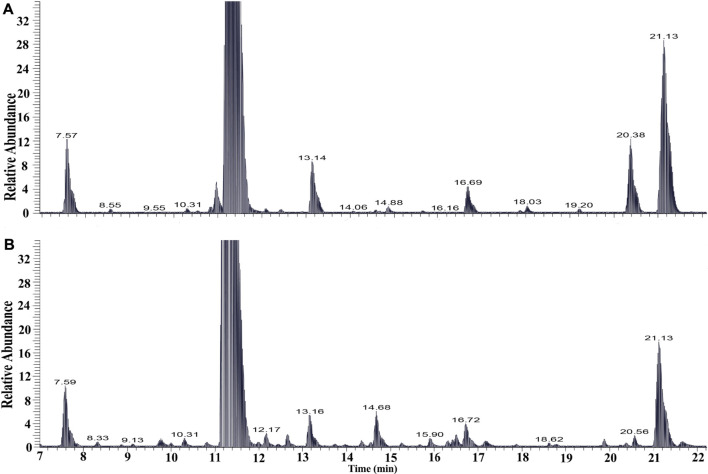
The base peak intensity chromatograms of samples *raw Reynoutria multiflora* Thunb. (RRM, **A)** and processed *Reynoutria multiflora* Thunb. (PRM for 24 h, **B)** derived from UHPLC-Q-Exactive plus orbitrap MS/MS (7–22 min).

The chemical profiles of RRM and processing PRMs were analyzed by UHPLC-Q-Exactive plus orbitrap MS/MS, the representative base peak chromatograms of RRM and processing PRM (24 h) are shown in Figure 1. Some differences were observed between the two base peak chromatograms. The stilbene glycosides and their derivatives were distributed from 7 to 22 min. The representative base peak chromatograms of processing of PRM (7–22 min) are shown in Figure 2.

### 3.2 Fragmentation Pathway of THSG and Derivatives

**FIGURE 3:**
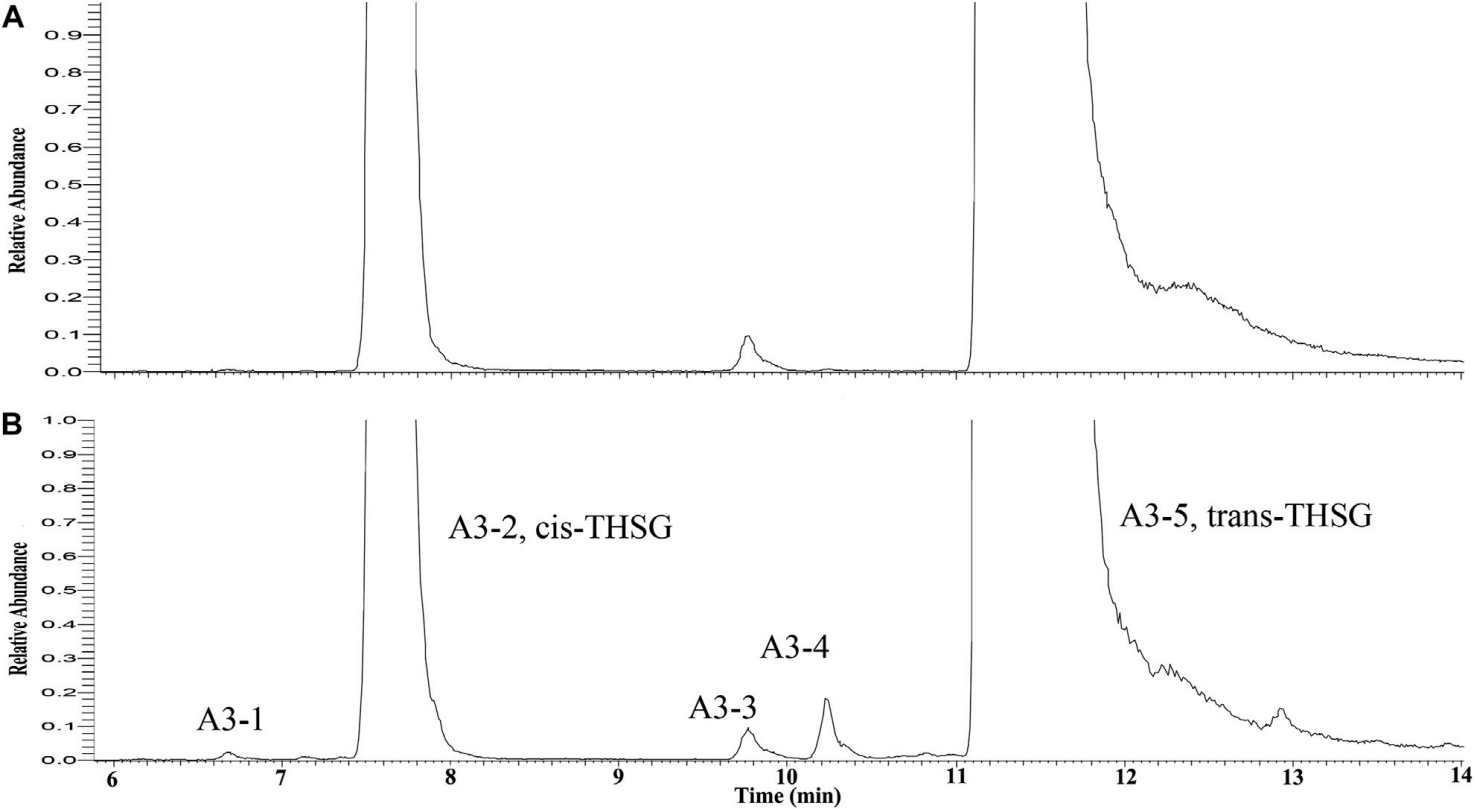
The extracted ion chromatograms of ion at *m/z* = 405.1187 **(A** *: raw Reynoutria multiflora* Thunb. RRM)*,* **B:** processed *Reynoutria multiflora* Thunb. (PRM 24 h)).

To identify the derivatives of THSG in the processing RM, the trans-THSG and cis-THSG standard were firstly analyzed by UPLC-Q-Exactive plus MS/MS under the above-mentioned conditions. Trans-THSG (**A3-5**, t_R_ = 11.18 min) and cis-THSG (**A3-2**, t_R_ = 7.58 min) had a [M-H]^−^ ion at *m/z* 405.1187 with only a dominant ion at *m/z* 243.0654 (C_14_H_11_O_4_) in MS^2^ spectrum. These two ions could be used as a diagnostic ion for identify stilbene glycosides. Compound **A3-1, A3-3 and A3-4** (t_R_ = 6.67, 9.75 and 9.89 min) also had an [M-H]^−^ ion at *m/z* 405.1187 (C_20_H_21_O_9_), and showed a fragment ion at *m/z* 243.0654 in their MS^2^ spectrum, indicating that they are isomers of THSG. **A3-5** was identified as trans-THSG and **A3-2** was cis-THSG, and **A3-1** should be isomer of cis-THSG, **A3-3 and A3-4** should be isomers of trans-THSG. (Figure 3).

### 3.3 Identification of Tetrahydroxystilbene-O-Hexoside Derivatives

During the processing, Maillard reaction occurred, producing a large number of compounds, including acetone alcohol, 2, 3-butanediol, succinic acid, 2, 3-dihydro-3, 5-dihydroxy-6-methyl-4H-pyranone (DDMP), 5-hydroxymethyl furfural (5-HMF) and its derivatives (Liu, 2018). Stilbene glycosides may react with products of Maillard reaction or small moleculars, such as gallic acid and catechuic acid, in high temperature and high humidity environment.

Most stilbene glycosides in RM showed common fragmentation pathways and two diagnostic fragment ions at *m/z* 405.1192(C_20_H_21_O_9_) and 243.0654 (C_14_H_11_O_4_). These were used for rapidly extracting and analyzing unknown stilbene glycosides. According to the structural characteristics of THSG, the linking points of stilbene derivatives with other compounds contain glycosyl hydroxyl moiety and phenyl hydroxyl moiety. According to the cleaved fragments, it can be inferred as follows: 1. if there is a fragment from loss of C_6_H_10_O_5_ by parent ion, the linking point should be phenyl hydroxyl moiety; 2. The cleavage fragment contains the ion at *m/z* 405.1187 of THSG and the ion at *m/z* (hexoside + substituent), and there is no fragment to loss of C_6_H_10_O_5_, so the linking point should be glycosyl hydroxyl moiety; 3. if the fragment is only the ion at *m/z* 243.0654, most of the glycosyl hydroxyl moiety may be linked, but there is also a probability that the hexoside of THSG and the substituent on the phenyl hydroxyl moiety will split at the same time, so the linking point cannot be determined in this case. We use tetrahydroxystilbene-O- (substituent)-hexoside to name them.

Compounds **A1-1** and **A1-2** displayed a [M-H]^−^ ion at *m/z* 375.1081 (C_19_H_19_O_8_) and the product ion at *m/z* 243.0654 derived from the loss of a pentose (mostly arabinose). By comparing with literature, Compounds **A1-1** and **A1-2** were tentatively identified as tetrahydroxystilbene-O-pentose.

Compounds **A2-1, A2-2**, and **A2-3** gave a [M-H]^−^ ion at *m/z* 389.1242 (C_20_H_21_O_8_) and the product ion at *m/z* 243.0654 derived from the loss of a deoxyhexose (mostly rhamnose), indicated that it was a THSG derivative. Compounds **A2-1, A2-2**, and **A2-3** were tentatively characterized as tetrahydroxystilbene-O-deoxyhexoside.

Compounds **A4-1** and **A4-2** displayed a high resolution [M-H]^−^ ion at *m/z* 423.1295 and gave element composition of C_20_H_23_O_10_. The MS^2^ spectra gave identical ions at *m/z* 261.0764 (C_14_H_13_O_5_) and 243.0654 (C_14_H_11_O_4_), respectively. The loss of C_6_H_10_O_5_ (hexoside) and H_2_O to produce the deprotonated moiety ion at *m/z* 243.0655, indicated can be identified as stilbene derivatives, but the specific structure is not yet determined.

Compounds **A5-1** ∼ **A5-4** showed the same [M-H]^−^ ion at *m/z* 433.1136 (C_21_H_21_O_10_) and the MS^2^ spectra gave ions at *m/z* 271.0608 (C_15_H_11_O_5_) and 243.0654 (C_14_H_11_O_4_). Without further information, compounds **A5-1** ∼ **A5-4** were tentatively characterized as tetrahydroxystilbene-O-hexoside-O-formic acid acyl (phenolic hydroxyl moiety).

Compounds **A6-1** and **A6-2** showed the same [M-H]^−^ ion at *m/z* 437.1450 (C_21_H_25_O_10_) and the MS^2^ spectra gave ions at *m/z* 275.0922 (C_15_H_15_O_5_) and *m/z* 243.0655 (C_14_H_11_O_4_), the loss of C_6_H_10_O_5_ (hexoside) and CH_4_O to produce the deprotonated moiety ion at *m/z* 243.0655, allowed us to infer that they were tetrahydroxystilbene derivative, but the specific structure is not yet determined.

Compounds **A7-1** ∼ **A7-5** gave a [M-H]^−^ ion at *m/z* 447.1300 (C_22_H_23_O_10_) and loss 204 Da to produce ion at *m/z* 243.0656 in the MS^2^ spectrum, which indicated that the presence of a hexose group and an acetyl. Thus, compounds **A7-1**∼ **A7-5** were preliminarily characterized as tetrahydroxystilbene-O-(acetyl)-hexoside.

Compounds **A8-1** and **A8-2** showed the same [M-H]^−^ ion at *m/z* 449.1086 (C_21_H_21_O_11_) and the MS^2^ spectra gave ions at *m/z* 287.0554 (C_15_H_11_O_6_) and 243.0654 (C_14_H_11_O_4_) form continuous loss of C_6_H_10_O_5_ and CO_2_. Thus, the carbonate acyl substituted THSG was detected and compounds **A8-1** and **A8-2** were identified as tetrahydroxystilbene-O-hexoside-O-carbonate acyl (phenolic hydroxyl moiety).

Compound **A9** displayed a high resolution [M-H]^−^ ion at *m/z* 457.1116 and gave element composition of C_23_H_21_O_10_, the product ion at *m/z* 243.0654 originated from the loss of C_9_H_10_O_6_ (hexoside + hydroxycyclopropenon). By investigating literature, compound **A9** was preliminarily identified as tetrahydroxystilbene-O-(hydroxycyclopropenon)-hexoside. Similarly, compounds **A10** and **A11** were tentatively identified as tetrahydroxystilbene-O-(acrylic acid acyl)-hexoside and tetrahydroxystilbene-O-(propionyl)-hexoside, since the loss of C_9_H_12_O_6_ (hexoside + acrylic acid) and C_9_H_14_O_6_ (hexoside + propionic acid) were detected.

Compounds **A12-1** and **A12-2** showed the same [M-H]^−^ ion at *m/z* 463.1244 (C_22_H_23_O_11_) and the MS^2^ spectra gave ion at *m/z* 243.0654 (C_14_H_11_O_4_) form loss of C_8_H_12_O_7_ (C_6_H_10_O_5_ and C_2_H_2_O_2_). Thus, the glycolic acid substituted THSG was detected and compounds **A12-1** and **A12-2** were identified as tetrahydroxystilbene-O- (glycolic acid acyl)-hexoside.

Compounds **A13-1**, **A13-2**, and **A13-3** showed the same [M-H]^−^ ion at *m/z* 477.1396 (C_23_H_25_O_11_) and the MS^2^ spectra gave ions at *m/z* 405.1184 (C_20_H_21_0_9_), 315.0859 (C_17_H_15_O_6_), 297.0763 (C_17_H_13_O_5_) and 243.0655 (C_14_H_11_O_4_). The ion at *m/z* 477.1396 loss of C_3_H_4_O_2_ produce the ion at *m/z* 405.1184, By comparing literature, compounds **A13-1**, **A13-2**, and **A13-3** were tentatively identified as tetrahydroxystilbene-O-hexoside-O-lactic acid acyl (phenolic hydroxyl moiety).

Compounds **A14-1** ∼ **A14-4** showed the same [M-H]^−^ ion at *m/z* 489.1759 (C_25_H_29_O_10_) and the MS^2^ spectra gave identical ions at *m/z* 405.1176 (C_20_H_21_0_9_), 327.1222 (C_19_H_19_O_5_) and 243.0656 (C_14_H_11_O_4_). The loss of C_5_H_8_O to produce the deprotonated THSG moiety ion at *m/z* 405.1176, Furthermore, the ion at *m/z* 327.1222 assigned as loss of C_6_H_10_O_5_ form the *m/z* 489.1759. By investigating literatures, compounds **A14-1** ∼ **A14-4** were identified as tetrahydroxystilbene-O-hexoside-O-valerate acyl (phenolic hydroxyl moiety).

Compound **A15** displayed a high resolution [M-H]^−^ ion at *m/z* 499.1241 and gave element composition of C_25_H_23_O_11_, the product ions at *m/z* 337.0704 (C_19_H_13_O_6_), 293.0812 (C_18_H_13_O_4_) and 243.0654 (C_14_H_11_O_4_) originated from the consecutive loss of C_6_H_10_O_5_ (hexoside), CO_2_ and C_4_H_2_ (5-hydroxyfuran-2-carbaldehyde). By investigating literature, compound **A15** was preliminarily identified as tetrahydroxystilbene-O-hexoside-O-5-hydroxyfuran-2-carbaldehyde (phenolic hydroxyl moiety).

**FIGURE 4:**
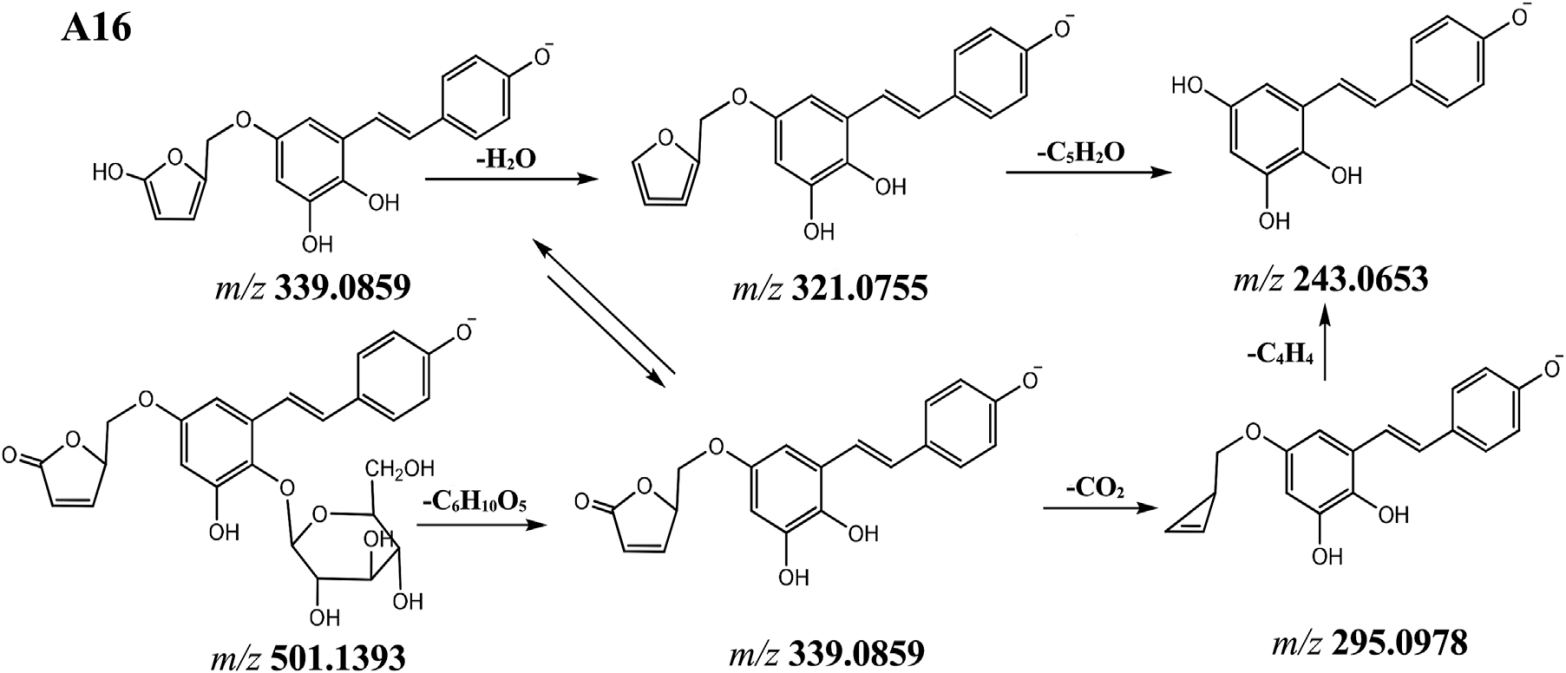
The proposal fragmentation pathway of compound of A16.

Compounds **A16-1** and **A16-2** showed the same [M-H]^−^ ion at *m/z* 501.1393 (C_25_H_25_O_11_) and the MS^2^ spectra gave ions at *m/z* 339.0858, 321.0756 and 243.0654 form continuous loss of C_6_H_10_O_5_ (hexoside), CO_2_ and C_4_H_4_ (Figure 4). By investigating literature, compounds **A16-1** and **A16-2** were identified as tetrahydroxystilbene-O-hexoside-O-4-hydroxymethyl-5H-furan-2-one (phenolic hydroxyl moiety).

Compound **A17** gave a [M-H]^−^ ion at *m/z* 503.1553 (C_25_H_27_O_11_) and the product ions at *m/z* 341.1019 (C_19_H_17_O_6_), 297.1125 (C_18_H_17_O_4_) and 243.0656 (C_14_H_11_O_4_). By comparing literature, compound **A17** was tentatively characterized as tetrahydroxystilbene-O-hexoside-O- 5-hydroxymethyl-4, 5-dihydrofuranone (phenolic hydroxyl moiety).

Compounds **A18-1** ∼ **A18-5** showed the same [M-H]^−^ ion at *m/z* 505.1346 (C_24_H_25_O_12_) and in **A18-1** and **A18-2** MS^2^ spectra, gave ions at *m/z* 405.1178 and *m/z* 243.0655, in **A18-3** ∼ **A18-5** MS^2^ spectra, gave ions at *m/z* 343.0799 and *m/z* 243.0655. By comparing literature, compounds **A18-1** and **A18-2** were preliminarily characterized as tetrahydroxystilbene-O-(succinic acid acyl)-hexoside, and compounds **A18-3** ∼ **A18-5** were identified as tetrahydroxystilbene-O-hexoside-O-succinic acid acyl (phenolic hydroxyl moiety).

Compounds **A19-1** and **A19-2** were eluted at 11.85 and 12.00 min, and the molecular formula was C_24_H_27_O_12_ (*m/z* 507.1500). The MS^2^ spectra gave identical ions at *m/z* 345.0966 (C_18_H_17_O_7_), *m/z* 313.0709 (C_17_H_13_O_6_), *m/z* 285.0763 (C_16_H_13_O_5_), *m/z* 255.0656 (C_15_H_11_O_4_) and *m/z* 243.0654 (C_14_H_11_O_4_). By comparing literature, compounds **A19-1** and **A19-2** were identified as tetrahydroxystilbene-O-hexoside-O-dihydroxybutyrate (phenolic hydroxyl moiety).

Compound **A20** gave a [M-H]^−^ ion at *m/z* 511.1603 (C_27_H_27_O_10_) and the product ions at *m/z* 349.1068 (C_21_H_17_O_5_) and 243.0655 (C_14_H_11_O_4_) form continuous loss of C_6_H_10_O_5_ (hexoside) and C_7_H_6_O (salicyloyl). By comparing literature, compound **A20** was tentatively characterized as tetrahydroxystilbene-O-hexoside-O-salicyloyl (phenolic hydroxyl moiety).

Compounds **A21-1** and **A21-2** showed the same [M-H]^−^ ion at *m/z* 512.1555 (C_26_H_26_O_10_N) and the MS^2^ spectra gave ions at *m/z* 405.1175 (C_20_H_21_O_9_) and 243.0655 (C_14_H_11_O_4_). By comparing literature, compounds **A21-1** and **A21-2** were tentatively identified as tetrahydroxystilbene-O-(aminocatecholoyl)-hexosides. Similarly, compound **A25** was tentatively identified as tetrahydroxystilbene-O-(pyroglutamyl)-hexoside.

**FIGURE 5:**
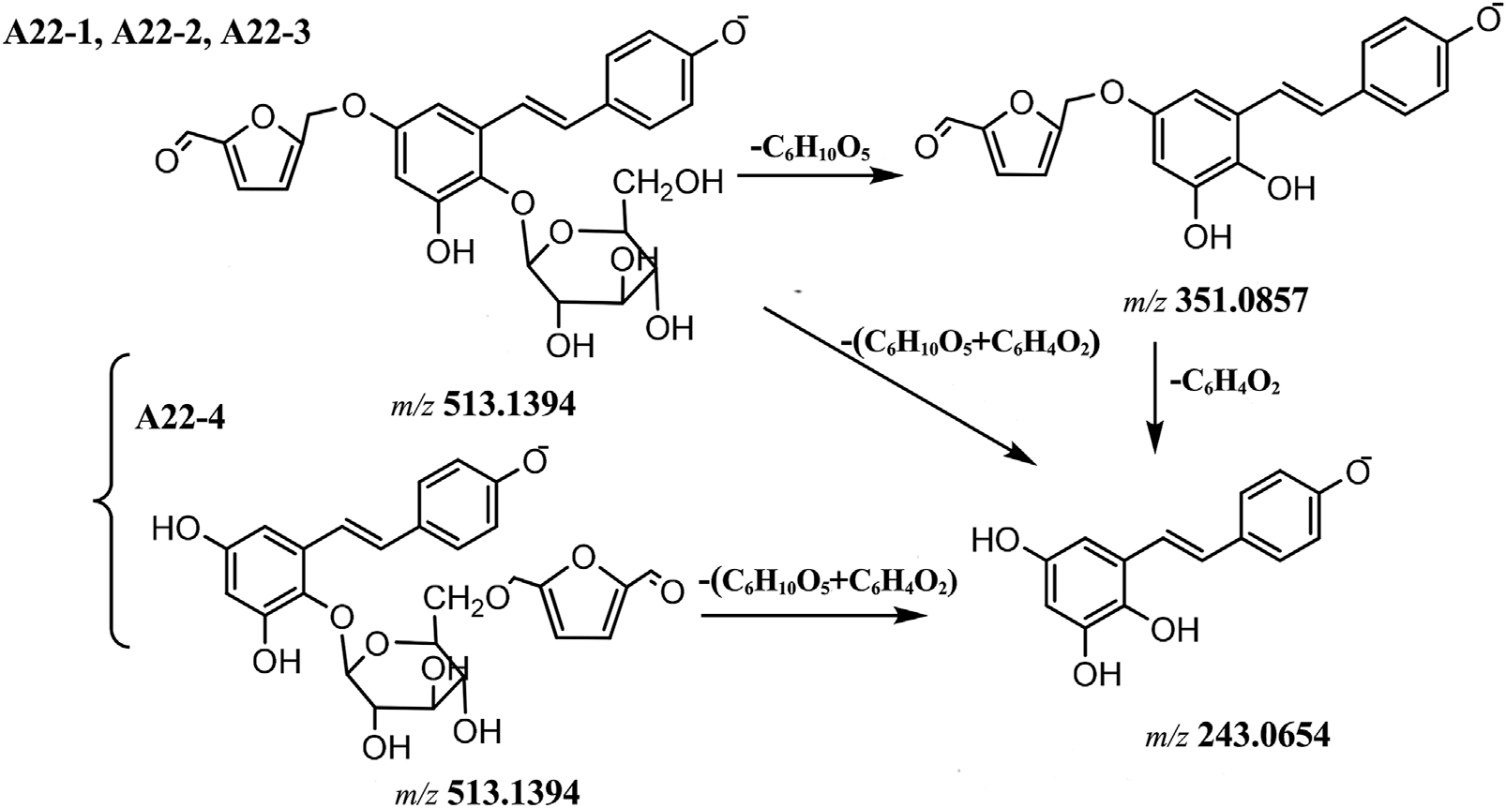
The proposal fragmentation pathway of compounds of A22.

Compounds **A22-1** ∼ **A22-4** showed the same [M-H]^−^ ion at *m/z* 513.1497 (C_26_H_25_O_11_), compounds **A22-1**, **A22-2** and **A22-3** loss 162 Da to produce ion at *m/z* 351.0862, and then loss 108 Da (C_6_H_4_O_2_) to produce ion at *m/z* 243.0656 in the MS^2^ spectrum. And in compound **A22-4** MS^2^ spectra, there was a fragment ion at *m/z* 243.0655, the proposal fragmentation pathway shown in Figure 5. By investigating literatures, compounds **A22-1**, **A22-2**, and **A22-3** were identified as tetrahydroxystilbene-O-hexoside-O-5-HMF (phenolic hydroxyl moiety), **A22-4** was identified as tetrahydroxystilbene-O-(5-HMF)-hexoside.

Compounds **A23-1** and **A23-2** were eluted at 15.29 and 15.69 min, and the molecular formula was C_26_H_27_O_11_ (*m/z* 515.1555). In addition, the product ions at *m/z* 353.1021 (C_20_H_17_O_6_) and *m/z* 243.0654 (C_14_H_11_O_4_) originated from the consecutive loss of hexoside (C_6_H_10_O_5_) and C_6_H_6_O_2_ (2, 5-bis-hydroxymethyl furan). By comparing literature, compounds **A23-1** and **A23-2** identified as tetrahydroxystilbene-O-hexoside-O-2, 5-bis-hydroxymethyl furan (phenolic hydroxyl moiety). And compound **A24** displayed a high resolution [M-H]^−^ ion at *m/z* 515.1179 and gave element composition of C_25_H_23_O_12_, the loss of C_5_H_2_O_3_ and C_6_H_10_O_5_ to produce the deprotonated moiety ion at *m/z* 243.0655. By investigating literatures, compound **A24** was tentatively characterized as tetrahydroxystilbene-O-(5-hydroxyfuran-2-carboxylic acid)-hexoside.

Compounds **A26-1** ∼ **A26-4** showed the same [M-H]^−^ ion at *m/z* 519.1495 (C_25_H_27_O_12_). In **A26-1** MS^2^ spectra, the fragment ion at *m/z* 243.0655, in **A26-2**, **A26-3** and **A26-4** MS^2^ spectra, the fragment ions at *m/z* 405.1167 (C_20_H_21_O_9_), 357.0967 (C_19_H_17_O_7_), 339.0855 (C_19_H_15_O_6_), 297.0760 (C_17_H_13_O_5_) and 243.0655 (C_14_H_11_O_4_), by investigating literatures, compound **A26-1** was identified as tetrahydroxystilbene-O-(glutaryl)-hexoside, **A26-2**, **A26-3** and **A26-4** were identified as tetrahydroxystilbene-O-hexoside-O-glutaryl (phenolic hydroxyl moiety).

Compounds **A27-1** (t_R_ = 12.84 min), **A27-2** (t_R_ = 13.07 min) and **A27-3** (t_R_ = 13.82 min) showed the same [M-H]^−^ ion at *m/z* 521.1294 (C_24_H_25_O_13_) and the MS^2^ spectra gave identical ions at *m/z* 405.1177 (C_20_H_21_O_9_), 359.1115 (C_19_H_19_O_7_) and 243.0754 (C_14_H_11_O_4_). The loss of C_4_H_4_O_4_ to produce the deprotonated THSG moiety ion at *m/z* 405.1177, thus, compounds **A27-1**, **A27-2**, and **A27-3** identified as tetrahydroxystilbene-O-hexoside-O-malic acid acyl (phenolic hydroxyl moiety).

Compounds **A28-1** ∼ **A28-5** showed the same [M-H]^−^ ion at *m/z* 525.1398 (C_27_H_25_O_11_), the MS^2^ of **A28-1** ∼ **A28-3** spectra gave ions at *m/z* 525.1398, 405.1179, 363.0883, 243.0858 and 137.0228. The product ions at *m/z* 363.0883 originated from the loss of hexoside (C_8_H_10_O_5_). Thus, the salicylic acid acyl substituted THSG was detected and compounds **A28-1** ∼ **A28-3** identified as tetrahydroxystilbene-O-hexoside-O-salicylic acid acyl (phenolic hydroxyl moiety). The MS^2^ of **A28-4** and **A28-5** spectra gave ions 405.1179, 243.0858 and 137.0228, but there were no 363.0833 fragment ion. Thus, compounds **A28-4** and **A28-5** were identified as tetrahydroxystilbene-O- (salicylic acid acyl)-hexoside.

Compounds **A29-1**, **A29-2** and **A29-3** gave a [M-H]^−^ ion at *m/z* 527.1190 (C_26_H_23_O_12_) and the MS^2^ spectra showed identical ions at *m/z* 365.0652 (C_20_H_13_O_7_) and 243.0659 (C_14_H_11_O_4_). The MS^2^ spectrum showed losses of C_6_H_10_O_5_ and C_6_H_2_O_3_, respectively, to produce characteristic aglycone ion at *m/z* 243.0659. By comparing literature, compounds **A29-1**, **A29-2**, and **A29-3** were tentatively identified as tetrahydroxystilbene-O-hexoside-O-5-formylfuran-2-carboxylyl (phenolic hydroxyl moiety).

**FIGURE 6:**
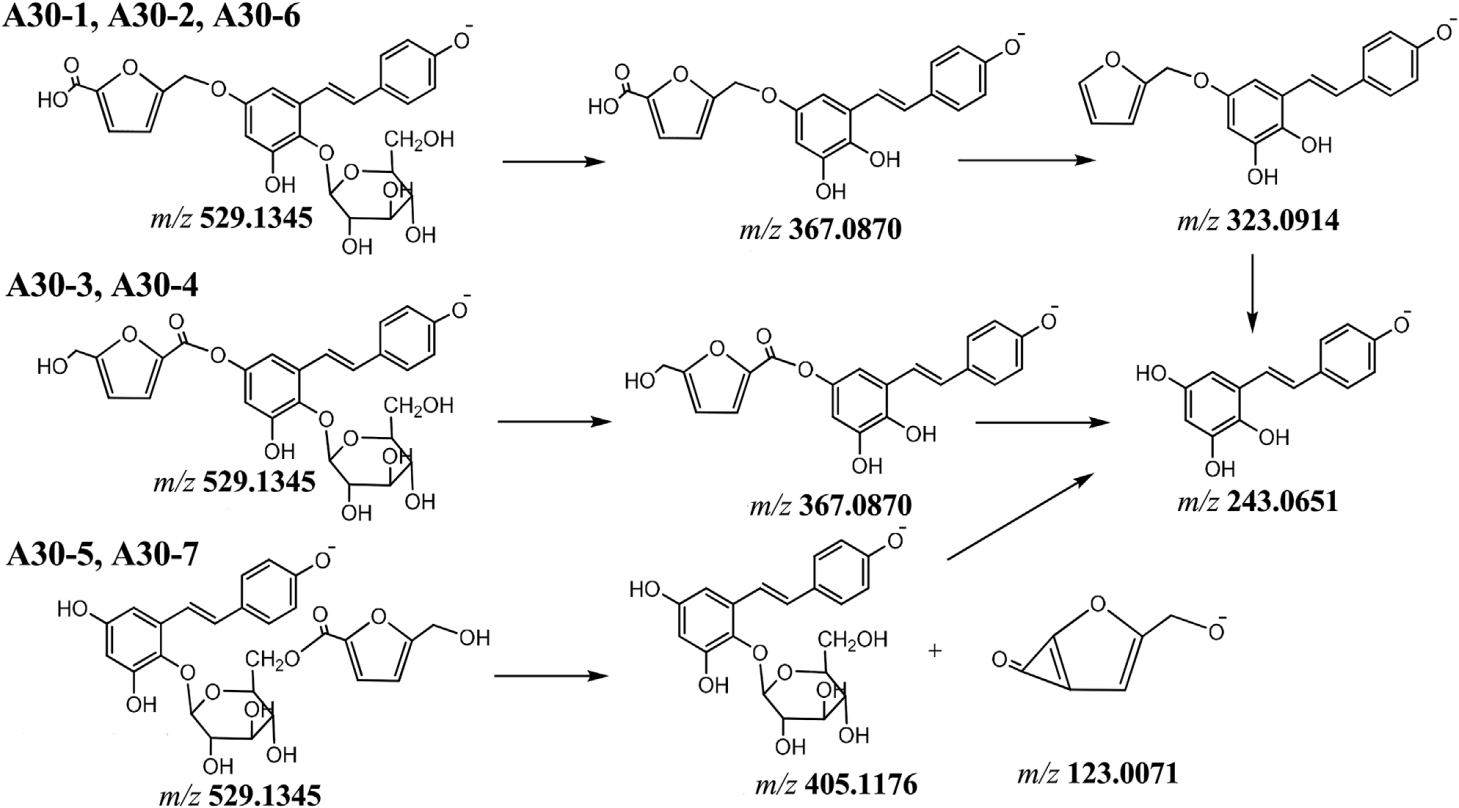
The proposal fragmentation pathway of compounds of A30.

Compounds **A30-1** ∼ **A30 ∼ 7** showed the same [M-H]^−^ ion at *m/z* 529.1345 (C_26_H_25_O_12_), the MS^2^ spectra of **A30-1**, **A30-2** and **A30-6** gave ions at *m/z* 367.0870 (C_20_H_15_O_7_), 323.0914 (C_19_H_15_O_5_) and 243.0651 (C_14_H_11_O_4_). The product ions originated from the consecutive loss of hexoside (C_6_H_10_O_5_), CO_2_ and C_5_H_4_O. In **A30-3** and **A30-4** spectra, gave ions at *m/z* 367.0807 (C_20_H_15_O_7_), 243.0656 (C_14_H_11_O_4_) and 123.0071 (C_6_H_3_O_3_), and in **A30-5** and **A30-7** spectra, gave ions at *m/z* 405.1176 (C_20_H_21_O_9_), 243.0656 (C_14_H_11_O_4_) and 123.0071 (C_6_H_3_O_3_). By investigating literatures, the substituent group of the compound was 5-hydroxymethyl-furfural. And according to the fragmentation fragments, it can be inferred that the binding sites are different (Figure 6). **A30-1, A30-2**, and **A30-6** were tentatively identified as tetrahydroxystilbene (phenolic hydroxyl moiety)-O-5-hydroxymethylfuran-2-carboxylyl-hexosides (hydroxyl moiety), **A30-3** and **A30-4** were identified as tetrahydroxystilbene (phenolic hydroxyl moiety)-O-5-hydroxymethylfuran- 2-carboxylyl-hexoside (carboxyl moiety), **A30-5** and **A30-7** were identified as tetrahydroxystilbene-O-(5-hydroxymethylfuran-2-carboxylyl)-hexoside.

Compounds **A31-1** ∼ **A31-10** were eluted at 2.58, 2.75, 3.58, 4.66, 5.00 min 5.64, 12.14, 15.02, 15.36, 15.74 min, they both showed an accurate [M-H]^−^ ion at *m/z* 531.1511 (C_26_H_27_O_12_), which were 128 Da higher than that of THSG. In their MS^2^ spectra, the [M-H]^−^ ion showed fragment ions at *m/z* 405.1180 (C_20_H_21_O_9_), 369.0966 (C_20_H_17_O_7_), 351.0863 (C_20_H_15_O_6_), 319.0796 (C_18_H_13_O_5_), 295.0609 (C_17_H_11_O_5_), and 243.0655 (C_14_H_11_O_4_). All the MS^2^ of the compounds have molecular fragment *m/z* 369.0966, indicated that the compounds are formed by dehydration of substituents and phenolic hydroxyl groups of stilbene glycosides. By investigating literature (Liu, 2018), compounds **A31-1** ∼ **A31-10** were tentatively characterized as tetrahydroxystilbene-O-hexoside-DDMP (phenolic hydroxyl moiety).

Compounds **A32-1** ∼ **A32-5** showed the same [M-H]^−^ ion at *m/z* 533.1658 (C_26_H_29_O_12_) and the MS^2^ spectra gave identical ions at *m/z* 371.1124 (C_20_H_18_O_7_), 327.0863 (C_18_H_16_O_6_) and 243.0657 (C_14_H_11_O_4_). The MS^2^ showed losses of C_6_H_3_O_3_ and C_6_H_10_O_5_ due to substituent and hexose moiety, respectively, to produce characteristic aglycone ion at *m/z* 243.0657 (C_14_H_11_O_4_). By comparing literature, compounds **A32-1** ∼ **A32-5** were tentatively characterized as tetrahydroxystilbene-O-hexoside-adipic acid acyl (phenolic hydroxyl moiety).

Compound **A33-1** ad **A33-2** gave a [M-H]^−^ ion at *m/z* 537.1609 (C_25_H_29_O_13_) and the product ion at *m/z* 243.0655 (C_14_H_11_O_4_) form loss of C_11_H_18_O_9_ (C_6_H_10_O_5_ and C_5_H_8_O_4_). By comparing literature, compound **A33-1** and **A33-2** were tentatively characterized as tetrahydroxystilbene-O-(arabinoyl)-hexoside.

Compounds **A34-1 ∼ A34-4** showed a [M-H]^−^ ion at *m/z* 541.1353 (C_27_H_25_O_12_) and the MS^2^ spectra of **A34-1** and **A34-2**, showed fragment ions at *m/z* 405.1175 (C_20_H_21_O_9_), 297.0610 (C_13_H_13_O_8_), 243.0657 (C_14_H_11_O_4_), 153.0179 (C_7_H_5_O_4_), respectively, indicated that they were THSG derivatives. The loss of C_7_H_5_O_4_ to produce the deprotonated THSG moiety ion at *m/z* 405.1175, allowed us to infer that they were protocatechuic acid substituted THSG products. And the ion at m/z 297.0610 assigned as protocatechuic acid acyl-hexoside moiety, produced protocatechuic acid ion at m/z 153.0179. **A34-3** and **A34-4** were eluted at 21.70 and 22.07 min, the [M-H]^−^ ions showed fragment ions at *m/z* 379.0793 (C_21_H_15_O_7_) and 243.0657 (C_14_H_11_O_4_), respectively, originated from the consecutive loss of hexoside (C_6_H_10_O_5_) and protocatechuic acid (C_7_H_5_O_4_). There results indicate that protocatechuic acid substituted to the phenolic hydroxyl moiety in compounds **A34-3** and **A34-4**. Therefore, compounds **A34-1** and **A34-2** were identified as tetrahydroxystilbene-O-hexoside-O-protocatechuic acid acyl (glycosyl hydroxyl moiety), and **A34-3** and **A34-4** were identified as tetrahydroxystilbene-O-hexoside-O-protocatechuic acid acyl (phenolic hydroxyl moiety).

Compound **A35** gave a [M-H]^−^ ion at *m/z* 543.1121 (C_26_H_33_O_13_) and the product ions at *m/z* 405.1196 (C_20_H_21_O_9_) and 243.0655 (C_14_H_11_O_4_) form consecutive loss of C_6_H_12_O_4_ and C_6_H_10_O_5_. By comparing literatures, compound **A35** was tentatively identified as tetrahydroxystilbene-O- (furan-dicarboxylic acid acyl)-hexoside. And compound **A36** gave a [M-H]^−^ ion at *m/z* 543.1501 (C_27_H_27_O_12_) and the product ion at *m/z* 381.0963 (C_21_H_17_O_7_), 337.1069 (C_20_H_17_O_5_) and 243.0655 (C_14_H_11_O_4_) form consecutive loss of C_6_H_10_O_5_, CO_2_ and C_6_H_6_O. By investigating literatures, **A36** was tentatively identified as tetrahydroxystilbene-O-hexoside-methoxymethyl-furancarboxylic acid acyl (phenolic hydroxyl moiety).

Compounds **A37-1** and **A37-2** showed the same [M-H]^−^ ion at *m/z* 547.1453 (C_26_H_27_O_13_) and the MS^2^ spectra gave ions at *m/z* 385.0914 (C_20_H_17_O_8_) and 243.0655 (C_14_H_11_O_4_) from consecutive loss of C_6_H_10_O_5_ and C_6_H_6_O_4_. By comparing literature, compounds **A37-1** and **A37-2** were tentatively identified as tetrahydroxystilbene-O-hexoside-oxoadipic acid acyl (phenolic hydroxyl moiety). Similarly, compounds **A38-1**, **A38-2** and **A38-3** were tentatively identified as tetrahydroxystilbene-O-hexoside-hydroxyadipic acid acyl (phenolic hydroxyl moiety), since the ions at *m/z* 387.1066 (C_20_H_19_O_8_) and 243.0655 (C_14_H_11_O_4_) from consecutive loss of C_6_H_10_O_5_ and C_6_H_8_O_4_.

Compounds **A39-1** ∼ **A39-5** displayed a high resolution [M-H]^−^ ion at *m/z* 551.1556 and gave element composition of C_29_H_27_O_11_. In **A39-2** ∼ **A39-5** MS^2^ spectra, the [M-H]^−^ showed fragment ions at *m/z* 405.1180 (C_20_H_21_O_9_), 243.0656 (C_14_H_11_O_4_), 163.0397(C_9_H_7_O_3_) and 145.0280 (C_9_H_5_O_2_). The product ions at *m/z* 405.1180 and 234.0656 originated from the consecutive loss of *p*-hydroxycinnamoyl (C_9_H_6_O_2_) and hexoside (C_6_H_10_O_5_), Thus, *p*-hydroxycinnamoyl substituted THSG was detected and compounds **A39-2** ∼ **A39-5** were identified as tetrahydroxystilbene-O-(*p*-hydroxycinnamoyl)-hexoside. In **A39-1** MS^2^ spectra, the [M-H]^−^ showed fragment ions at 399.1018 (C_23_H_17_O_6_, M-C_6_H_10_O_5_), 243.0655, 163.0396 and 145.0279, indicated *p*-hydroxycinnamoyl was linked to phenolic hydroxyl moiety. Thus, compound **A39-1** was identified as tetrahydroxystilbene-O-hexoside-*p*-hydroxycinnamoyl (phenolic hydroxyl moiety).

Compounds **A40-1** ∼ **A40-8** showed the same [M-H]^−^ ion at *m/z* 557.1295 (C_27_H_25_O_13_) and the MS^2^ spectra gave identical ions at *m/z* 405.1179 (C_20_H_21_O_9_), 313.0555 (C_13_H_13_O_9_), 243.0654 (C_14_H_11_O_4_) and 169.0127 (C_7_H_5_O_5_). The loss of C_6_H_10_O_5_ and C_7_H_4_O_4_ to produce the deprotonated resveratrol moiety ion at *m/z* 243.0654, a galloyl group was present, the ion at 313 ([galloylglucose—H]) appeared as base peak in the MS^2^ spectra. It could further fragment ion *m/z* 169 ([gallic acid – H]^−^). Therefore, compounds **A40-1** ∼ **A40-8** were identified as tetrahydroxystilbene-O-hexoside-O-galloyl (glycosyl hydroxyl moiety) (Qiu et al., 2013).

Compounds **41-1** and **41-2** showed the same [M-H]^−^ ion at *m/z* 561.1609 (C_27_H_29_O_13_) and the MS^2^ spectra gave identical ions at *m/z* 405.1174 (C_20_H_21_O_9_) and 243.0654 (C_14_H_11_O_4_). By comparing literatures, compounds **41-1** and **41-2** were identified as tetrahydroxystilbene-O- (gabosine C)-hexoside.

**FIGURE 7:**
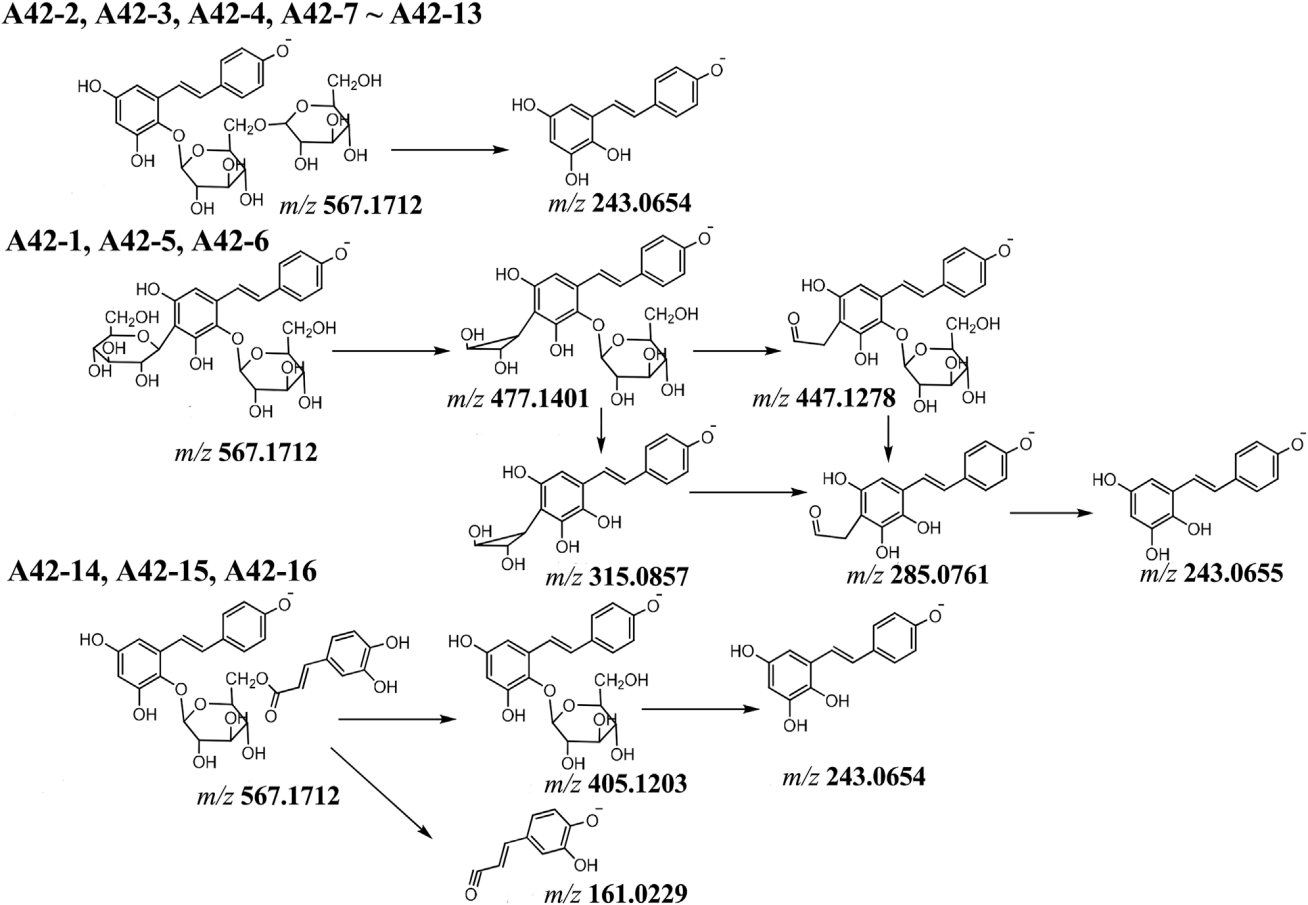
The proposal fragmentation pathway of compounds of A42.

Compounds **A 42-1 ∼ A 42-13** gave precursor ion [M-H]^−^ at *m/z* 567.1712 (C_26_H_31_O_14_), in **A42-2 ∼ A42-4** and **A42-7 ∼ A42-13** MS^2^ spectra, the [M-H]^−^ ion showed fragment ion at *m/z* 243.0654 (C_14_H_11_O_4_), indicated that the consecutive neutral loss of hexoside, they were disaccharide THSGs. By comparing literature, they were tentatively characterized as tetrahydroxystilbene-O-di-hexosides (Figure 7). But in **A42-1, A42-5**, and **A42-6** MS^2^ spectra, the [M-H]^−^ ion showed fragment ions at *m/z* 477.1401 (C_23_H_25_O_11_), 447.1278 (C_22_H_23_O_10_), 315.0857 (C_17_H_15_O_6_), 285.0761 (C_16_H_13_O_5_), and 243.0655 (C_14_H_11_O_4_), respectively. The fragment ions 477.1401 [M-H-90 Da, C_3_H_6_O_3_]^−^, 447.1278 [M-H-120 Da, C_4_H_8_O_4_]^−^ are diagnostic the last two neutral loss fragments suggested that a C-glycoside was connected with the stilbene glycoside. Therefore, **A42-1, A42-5** and **A42-6** were determined as tetrahydroxystilbene-O-hexoside-C-glycoside.

**A42-14** (t_R_ = 18.29 min)**, A42-15** (t_R_ = 19.50 min) and **A42-16** (t_R_ = 21.64 min) showed the molecular formula were C_29_H_27_H_12_ (*m/z* = 567.1504), which was 162 Da heavier than that of THSG, and different from **A42-1 ∼ A42-13**. In their MS^2^ spectra, the [M-H]^−^ ion showed fragment ions at *m/z* 405.1203 (C_20_H_21_O_9_), 243.0654 (C_14_H_11_O_4_), 161.0229 (C_9_H_5_O_3_), respectively. Indicated that the consecutive loss of caffeoyl (C_9_H_6_O_3_) and hexoside (C_6_H_10_O_5_). Thus, the caffeoyl substituted THSG was detected and compound **A42-14**, **A42-15** and **A42-16** identified as tetrahydroxystilbene-O-(caffeoyl)-hexoside.

Compounds **A43-1** ∼ **A43-6** showed the same [M-H]^−^ ion at *m/z* 573.1251 (C_27_H_25_O_14_) and the MS^2^ spectra gave identical ions at *m/z* 243.0657 (C_14_H_11_O_4_), 166.9971 (C_7_H_3_O_5_) and 123.0071 (C_6_H_3_O_3_). By comparing literature, Compounds **A43-1** ∼ **A43-6** were tentatively identified as tetrahydroxystilbene-O- (tetrahydroxybenzoic acid acyl)-hexoside.

Compound **A44** gave a [M-H]^−^ ion at *m/z* 575.1402 (C_27_H_27_O_14_) and the product ions at *m/z* 337.0707 (C_19_H_13_O_6_) and 244.0655 (C_14_H_11_O_4_). By comparing literatures, compound **A44** was tentatively identified as tetrahydroxystilbene-O- (dioxoheptane-dicarboxylic acid acyl)-hexoside.

Compounds **A45-1** ∼ **A45-5** showed the same [M-H]^−^ ion at *m/z* 581.1655 (C_30_H_29_O_12_), the MS^2^ spectra gave ions at *m/z* 405.1177 (C_20_H_21_O_9_), 337.0921(C_16_H_17_O_8_), 243.0655(C_14_H_11_O_4_), 193.0493 (C_10_H_9_O_4_) and 175.0387 (C_10_H_7_O_3_). Its MS^2^ spectrum gave characteristic ions at *m/z* 337.0921 ([feruoylglucose-H]^−^), *m/z* 193.0493 ([ferulic acid-H]^−^) and *m/z* 175.0387, therefore, compounds **A45-1** ∼ **A45-5** were identified as tetrahydroxystilbene-O-hexoside-O-feruloyl (glycosyl hydroxyl moiety).

Compound **A46** gave a [M-H]^−^ ion at *m/z* 591.2075 (C_29_H_35_O_13_) and the product ions at *m/z* 405.1203 (C_20_H_21_O_9_), 243.0655 (C_14_H_11_O_4_) and 185.0806 (C_9_H_13_O_4_). By comparing literature, compound **A46** was tentatively identified as tetrahydroxystilbene-O- (hydroxynonanedioic acid acyl)-hexoside.

Compound **A47** gave a [M-H]^−^ ion at *m/z* 613.1769 (C_27_H_33_O_16_) and the product ions at *m/z* 405.1203 (C_20_H_21_O_9_) and 243.0655 (C_14_H_11_O_4_). By comparing literatures, compound **A47** was tentatively identified as tetrahydroxystilbene-O-(Glucoheptanoyl)-hexoside.

Compounds **A48-1**, **A48-2** and **A48-3** showed the same [M-H]^−^ ion at *m/z* 719.1825 (C_33_H_35_O_18_) and the MS^2^ spectra gave identical ions at *m/z* 557.1287 (C_27_H_25_O_13_), 405.1174 (C_20_H_21_O_9_), 313.0557 (C_13_H_13_O_9_), 243.0655 (C_14_H_11_O_4_) and 169.0126 (C_7_H_5_O_5_), the loss of C_6_H_10_O_5_ to produce compound **A39** ion at *m/z* 557.1287, and the fragmentation ions were the same. Therefore, **A48-1**, **A48-2** and **A48-3** were identified as tetrahydroxystilbene-O-dihexoside -galloyl (glycosyl hydroxyl moiety).

Compounds **A49-1** ∼ **A49-6** displayed a high resolution [M-H]^−^ ion at *m/z* 827.2399 and gave element composition of C_40_H_43_O_19_. The MS^2^ spectra gave ions at 405.1165 (C_20_H_21_O_9_), 259.0607 (C_14_H_11_O_5_) and 243.0656 (C_14_H_11_O_4_), forming the ions 405.1165 and 243.0656 indicated that they should be THSG derivatives. By comparing literature, compounds **A49-1** ∼ **A49-6** were tentatively identified as polygonumoside C/D.

**FIGURE 8:**
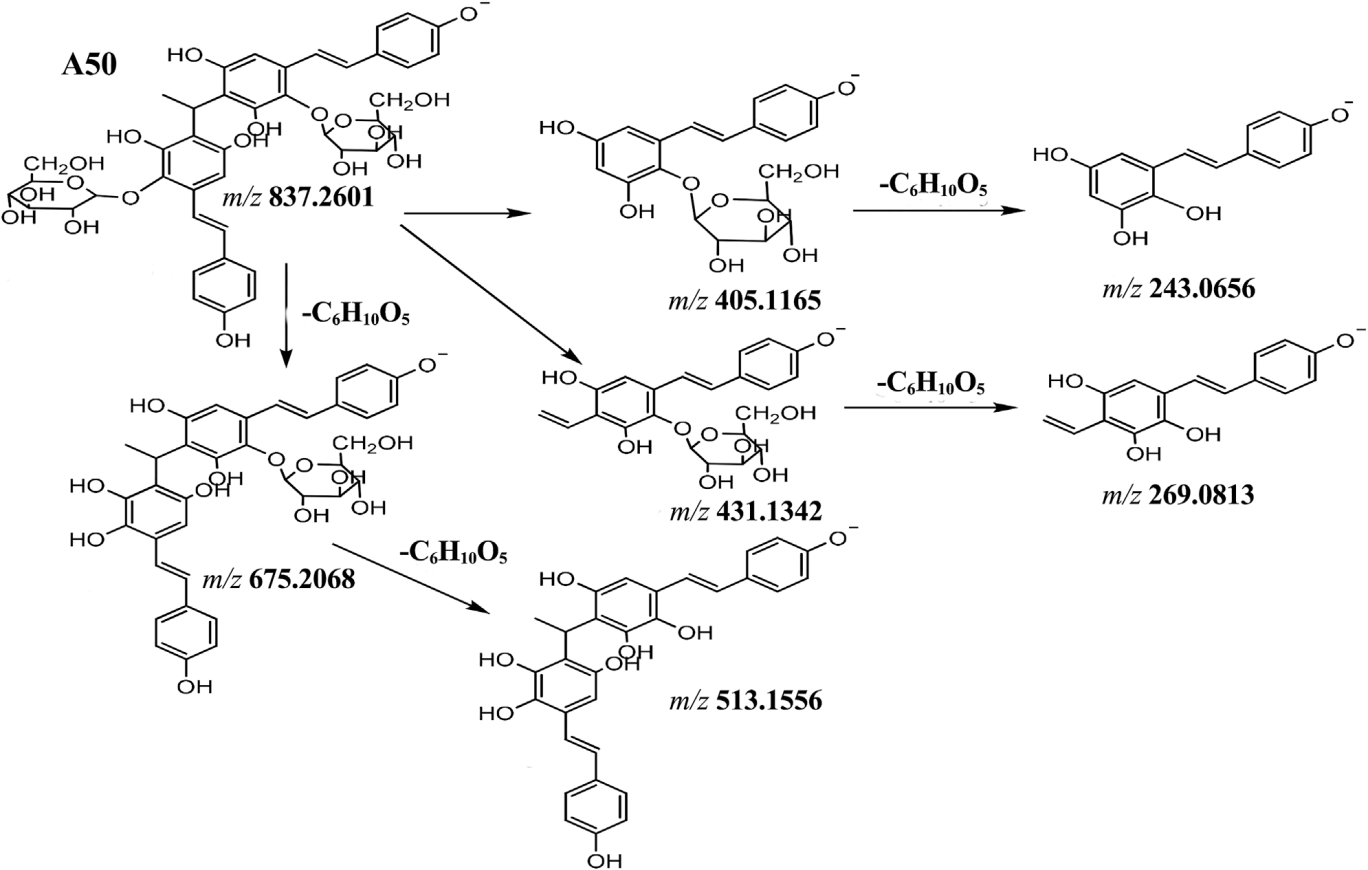
The proposal fragmentation pathway of compound of A50.

Compounds **A50-1** ∼ **A50-8** displayed a high resolution [M-H]^−^ ion at *m/z* 837.2601 and gave element composition of C_42_H_45_O_18_. The MS^2^ spectra gave ions at *m/z* 675.2068 (C_36_H_35_O_13_), 513.1556 (C_30_H_25_O_8_), 431.1342 (C_22_H_23_O_9_), 405.1165 (C_20_H_21_O_9_), 269.0813 (C_16_H_13_O_4_) and 243.0656 (C_14_H_11_O_4_). The consecutive neutral loss of hexoside, forming ions at *m/z* 675.2068 and 513.1556, and forming the ions 405.1165, 243.0656 and 431.1342, 269.0813 indicated that was cleavage into two glycosides. By comparing literature, compounds **A50-1** ∼ **A50-8** were identified as polygonumnolide D (Figure 8). Similarly, compound **A52** was tentatively characterized as hydroxylation polygonumnolide D, since the [M-H]^−^ ion at *m/z* 853.2560 (C_42_H_45_O_19_), which was 16 Da (O) higher than that of **A50**, and the MS^2^ spectra gave ions at *m/z* 447.1300 (C_22_H_23_O_10_), 405.1175 (C_20_H_21_O_9_), 285.0765 (C_16_H_13_O_5_) and 243.0656 (C_14_H_11_O_4_).

Compounds **A51-1** ∼ **A51-5** showed the same [M-H]^−^ ion at *m/z* 841.2551 (C_41_H_45_O_19_), which was 14 Da (CH_2_) higher than that of **A49**. The MS^2^ spectra gave ions at *m/z* 405.1182 (C_20_H_21_O_9_), 273.0764(C_15_H_13_O_5_) and 243.0655(C_14_H_11_O_4_), and the ion *m/z* 273.0764 was 14 Da (CH_2_) higher than that of **A49** ion *m/z* 259.0607. therefore, compounds **A51-1** ∼ **A51-5** were identified as methylation polygonumoside C/D. Similarly, compounds **A53-1 ∼ A53-4** were identified as hydroxylation methylation polygonumoside C/D. since the [M-H]^−^ ions at *m/z* 857.2502 (C_41_H_45_O_20_), which was 14 Da (CH_2_) higher than that of **A51**, and the MS^2^ spectra gave ions at *m/z* 405.1172 (C_20_H_21_O_9_), 289.0709 (C_15_H_13_O_6_) and 243.0656 (C_14_H_11_O_4_).

### 3.4 Identification of Trihydroxystilbene-O-Hexoside Derivatives

Compounds **B1-1**, **B1-2** and **B1-3** gave a [M-H]^−^ ion at *m/z* 389.1240 (C_20_H_21_O_8_) and the product ion at *m/z* 227.0702 (C_14_H_11_O_3_). The loss of C_6_H_10_O_5_ was confirmed by MS^2^ spectra and indicated a hexose neutral loss. Compared with the control substance, **B1-2** was identified as polydatin, compounds **B1-1** and **B1-3** were identified as isomer polydatin.

Compounds **B2-1** ∼ **B2-4** showed the same [M-H]^−^ ion at *m/z* 541.1345 (C_27_H_25_O_12_) and the MS^2^ spectra gave ions at *m/z* 313.0559 (C_13_H_13_O_9_), 227.0702 (C_14_H_11_O_3_), 169.0128 (C_7_H_5_O_5_). Similar with compounds A40, compounds **B2-1** ∼ **B2-4** were identified as trihydroxystilbene-O-hexoside-O-*galloyl* (glycosyl hydroxyl moiety).

Compounds **B3-1** and **B3-2** showed the same [M-H]^−^ ion at *m/z* 457.1136 (C_23_H_21_O_10_) and the MS^2^ spectra gave ions at *m/z* 295.0605 (C_17_H_11_O_5_) and 227.0702 (C_14_H_11_O_3_). By comparing literature, compounds **B3-1** and **B3-2** were identified as trihydroxystilbene-O-hexoside-O-acid deltique acyl (phenolic hydroxyl moiety).

Compound **B4** gave a [M-H]^−^ ion at *m/z* 535.1816 (C_26_H_31_O_12_) and the product ion at *m/z* 227.0702 (C_14_H_11_O_3_) derived from the loss of C_6_H_10_O_5_ (hexoside) and C_6_H_10_O_4_ (deoxyhexose, mostly rhamnose). By investigating literatures, compound **B4** was identified as trihydroxystilbene-(deoxyhexose)-O-hexoside.

Compound **B5** displayed a high resolution [M-H]^−^ ion at *m/z* 359.1132 and gave element composition of C_19_H_19_O_7_, the product ions at *m/z* 359.1129 (C_19_H_19_O_7_) and 227.0701 (C_14_H_11_O_3_). The product ion at *m/z* 227.0701 originated from the loss of pentose (mostly arabinose). Therefore, compound **B5** was identified as trihydroxystilbene-O-pentose.

### 3.5 Identification of Pentahydroxystilbene Glycoside Derivatives

Compounds **C1-1** ∼ **C1-7** displayed a high resolution [M-H]^−^ ion at *m/z* 421.1138, and gave element composition of C_20_H_21_O_10_, which was 14 Da (CH_2_) higher than that of THSG, the MS^2^ spectra gave ion at 259.0609 (C_14_H_11_O_5_). By comparing literature, compounds **C1-1** ∼ **C1-7** were tentatively identified as pentahydroxystilbene glycosides.

Compound **C2** gave a [M-H]^−^ ion at *m/z* 545.1291 (C_26_H_25_O_13_) and the product ions at *m/z* 421.1128 (C_20_H_21_O_10_), 259 (C_14_H_11_O_5_) and 123.0070 (C_6_H_3_O_3_) derived from the loss of C_6_H_5_O_5_ (5-HMF) and C_16_H_10_O_5_ (hexoside). By investigating literatures, compound **C2** was identified as pentahydroxystilbene-(5-HMF)-O-hexoside.

### 3.6 Identification of Tetrahydroxy-Phenanthrene-O-Hexoside Derivatives

Compounds **D1-1** and **D1-2** gave a [M-H]^−^ ion at *m/z* 403.1030 (C_20_H_19_O_9_) and prominent fragment ion at *m/z* 241.0497 (C_14_H_9_O_4_) in MS^2^ spectrum, which were showed 2 Da less than that of THSG. It can be inferred that they were dehydrogenated product of THSG. By comparing literatures (Qiu et al., 2013), compounds **D1-1** and **D1-2** were identified as tetrahydroxy-phenanthrene-O-hexoside.

Compounds **D2-1** ∼ **D2-8** showed the same [M-H]^−^ ion at *m/z* 549.1605 (C_26_H_29_O_13_) and the MS^2^ spectra gave ions at *m/z* 387.1072 (C_20_H_19_O_8_), 297.0760 (C_17_H_13_O_5_) and 241.0497 (C_14_H_9_O_4_). Similarly compounds **A38**, compounds **D2-1** ∼ **D2-8** were identified as tetrahydroxy-phenanthrene-O-hexoside-O-*p*-hydroxycinnamoyl (phenolic hydroxyl moiety).

### 3.7 Identification of Dihydrotetrahydroxystilbene-O-Hexoside Derivatives

Compound **E1** gave a [M-H]^−^ ion at *m/z* 407.1343 (C_20_H_23_O_9_) and prominent fragment ion at *m/z* 245.0811 (C_14_H_13_O_4_) in MS^2^ spectrum, which were showed 2 Da higher than that of THSG. It can be inferred that they were dihydrogenated product of THSG. By comparing literatures, compounds **E1** was identified as dihydrotetrahydroxystilbene-O-hexoside.

Compounds **E2-1** and **E2-2** showed the same [M-H]^−^ ion at *m/z* 527.1552 (C_27_H_27_O_11_) and the MS^2^ spectra gave ions at *m/z* 365.1017 (C_21_H_17_O_6_), 335.0918 (C_20_H_15_O_5_) and 245.0814 (C_14_H_13_O_4_). Similarly compounds **A27**, compounds **E2-1** and **E2-2** were identified as dihydrotetrahydroxystilbene-O-hexoside-salicylic acid acyl (phenolic hydroxyl moiety).

Compound **E3** displayed a high resolution [M-H]^−^ ion at *m/z* 539.1766, and gave element composition of C_25_H_31_O_13_. The MS^2^ spectra gave ion at 245.0811 (C_14_H_13_O_4_) derived from the loss of C_6_H_10_O_5_ (hexoside) and C_5_H_8_O_4_ (pentose, mostly arabinose). Compound **E3** was identified as dihydrotetrahydroxystilbene-O-(pentose)-hexoside.

### 3.8 Identification of Pentahydroxy-Phenanthrene-O-Hexoside

Compounds **F1-1** and **F1-2** showed the same [M-H]^−^ ion at *m/z* 419.0980 (C_20_H_19_O_10_) and the MS^2^ spectra gave ion at *m/z* 257.0542 (C_14_H_9_O_5_), which were showed 16 Da higher than that of compounds D1. Therefore, compounds **F1-1** and **F1-2** were identified as pentahydroxy-phenanthrene-O-hexosides.

### 3.9 Identification of Dihydroxystilbene-O-Hexoside Derivatives

Compound **G1** gave a [M-H]^−^ ion at *m/z* 373.1286 (C_20_H_21_O_7_) and prominent fragment ion at *m/z* 211.0751 (C_14_H_13_O_2_) in MS^2^ spectrum, which were showed 32 Da less than that of THSG. It can be inferred that they were dedihydroxylation product of THSG. Therefore, compounds **G1** was identified as dihydroxystilbene-O-hexoside.

Compounds **G2-1** and **G2-2** showed the same [M-H]^−^ ion at *m/z* 525.1396 (C_27_H_25_O_11_) and the fragment ions at *m/z* 525.1392, 313.0558 (C_13_H_13_O_9_), 211.0751 (C_14_H_13_O_2_), 169.0128 (C_7_H_5_O_5_) and 151.0020 (C_7_H_3_O_4_) in MS^2^ spectra. Similar with compounds **A39** and compounds **B2**, compounds **G2-1** and **G2-2** were identified as dihydroxystilbene-O-hexoside-O-galloyl (glycosyl hydroxyl moiety).

### 3.10 Structural Changes of Stilbene Glycosides

#### 3.10.1 Structural Changes of Parent Stilbene Glycosides

**FIGURE 9:**
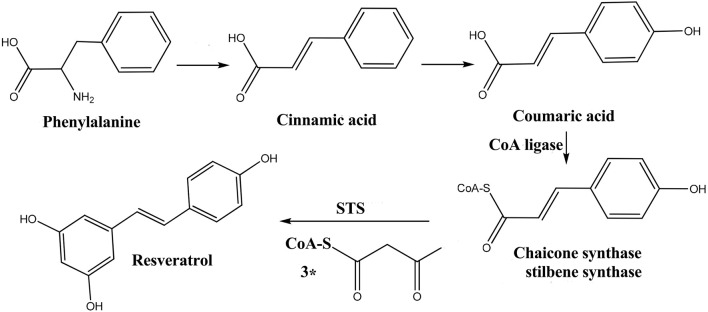
The biosynthesis pathway of resveratrol.

**FIGURE 10:**
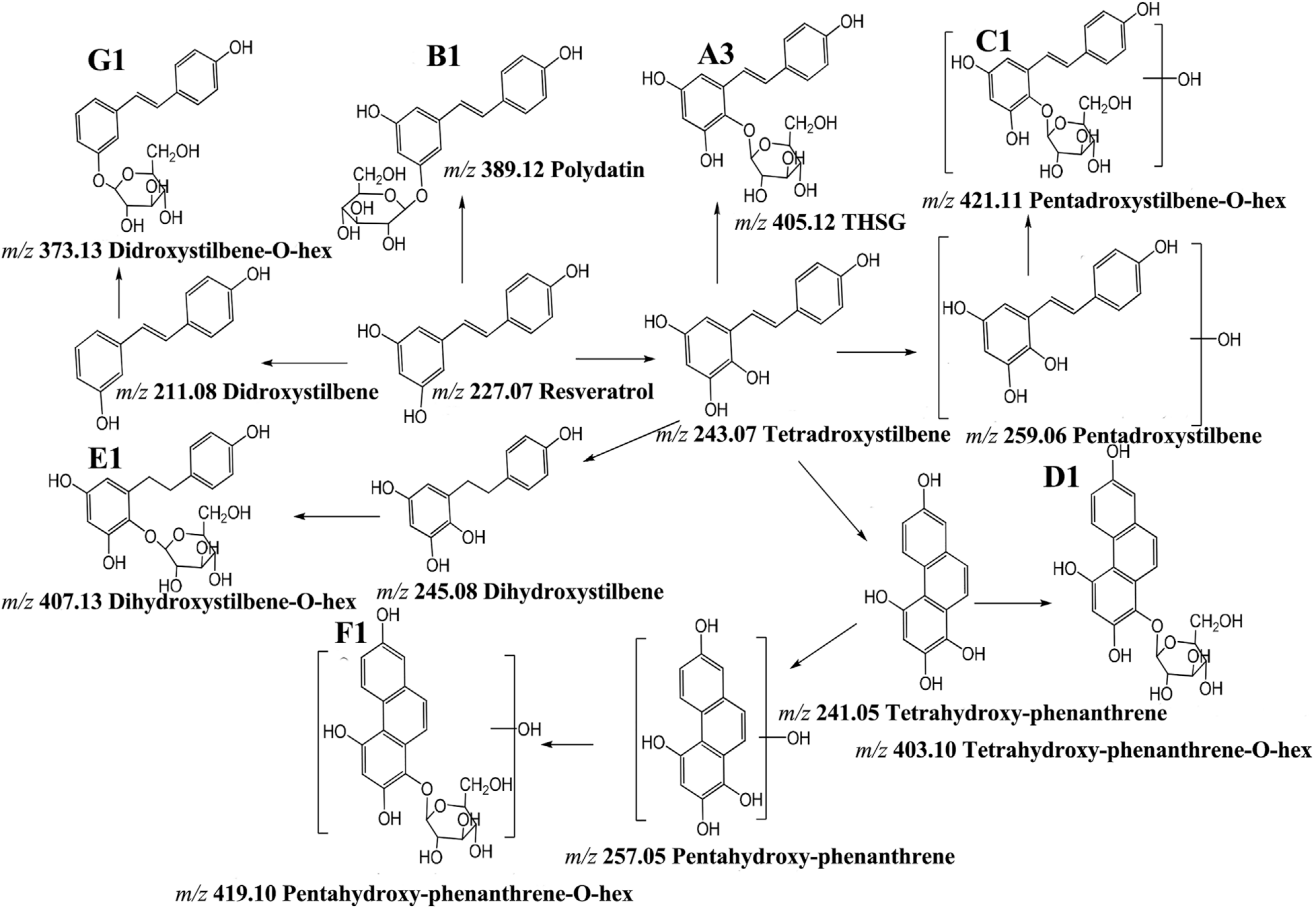
The structural changes pathway of stilbene glycosides.

Stilbenes are regared to be derived from phenylalanine metabolism in plants (Isvett et al., 2009) (Figure 9). THSG is the highest and most reported compound in RM Studies have shown that resveratrol was the intermediate product of THSG, which was hydroxylated to form terahydroxystilbene, and then glycosylated to form THSG (compounds **A3**) (Zhao, 2017). Polydatin (compound **B1-2**) was glycosylated form resveratrol. Pentahydroxystilben-O-hexoside (compounds **C1**) was synthesized by rehydroxylation of tetrahydroxystilbene and then glycosylation. The phenanthrene moiety was formed by cyclization of 6-H and 2′-H positions of tetrahydroxystilbene, which can stabilize the cyclization of aglycone, and then glycosylated to form tetrahydroxy-phenanthrene-O-hexoside (compounds **D1**). The tetrahydroxy-phenanthrene was hydroxylated to form pentahydroxy-phenanthrene, then glycosylated to form pentahydroxy-phenanthrene-O-hexoside (compounds **F1**). Dihydrotetrahydroxystilbene-O-hexoside (compounds **E1**) was synthesized from tetrahydroxystilbene by double bond opening and then glycosylated. The resveratrol lost a hydroxyl group in the plant, and dihydroxystilbene-O-hexoside (**G1**) was obtained by the glycosylation of dihydroxystilbene (Figure 10).

### 3.11 Structural Changes in Stilbene Glycosides Substituents

**FIGURE 11:**
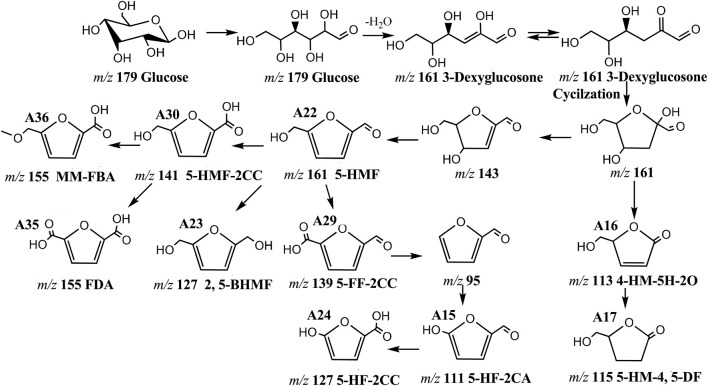
The structural changes pathway of 5-HMF.

Compounds containing malonyl, acetyl, caffeoyl and other aromatic acyls are very common in various plants (Barnes et al., 1994; Klejdus et al., 2001; Ye et al., 2007). And galloyl, malonyl, acetyl, caffeoyl, coumaroyl, feruloyl substituent have been reported in raw PM in our previous research (Qiu et al., 2013). In total, fifty-five substituents were found in this study. Except for the above-mentioned substituents, there was also some organic acids existed, such as formic acid, acetic acid, carbonic acid, propionic acid, glycolic acid, lactic acid, valeric acid, succinic acid, dihydroxy butyric acid, salicylic acid, glutaric acid, malic acid, catechuic acid and *p*-hydroxybenzoic acid. Maillard reaction products, mainly includes 2, 3-dihydro-3, 5-dihydroxy-6-methyl-4H-pyranone (DDMP, **A31**) and 5-hydromethylfurfura (5-HMF, **A22**) and its derivatives from glucose or glycine were also observed as stilbene glycosides substituents (Perez Locas and Yaylayan, 2008; Harishchandra et al., 2011; Liu, 2018). The aldehyde end of 5-HMF is oxidized to carboxylic acid, 5-hydroxymethylfuran-2-carboxyl acid (5-HMF-2-CC, **A30**) was produced, then methylation and oxidation produced methoxymethyl-furancarboxylic acid (MM-FBA, **A36**) and furan-dicarboxylic acid (FDA, **A35**). 2, 5-bis-(hydroxymethyl) furan (2, 5-BHMF, **A23**) was synthesized by the reduction of 5-HMF. 5-formylfuran-2-carboxyl acid (5-FF-2CC, **A29**) was synthesized by the oxidation of the hydroxyl end of 5-HMF. Then decarboxylation and reoxidation were carried out to form 5-hydroxyfuran-2-carbaldehyde (5HF-2CA, **A15**), then, 5-hydroxyfuran-2-carboxylic acid (5-HF-2CC, **A24**) was formed by oxidation of aldehyde end. 4-hydroxymethyl-5h-furan-2-one (4-HM-5H-2O, **A16**) was synthesized from 2, 4-dihydroxy-5- (hydroxymethyl) oxolane-2-carbaldehyde by dehydration and CH_2_O removal, then hydrogenation reaction was reacted to produce 5-hydroxymethyl-4,5-dihydrofuranone (5-HM-4, 5-DF, **A17**), and the change path of 5-HMF is shown in Figure 11) all substituents information is shown in Supplementary Table S1.

### 3.12 Content Change Trend of Stilbene Derivatives

The peak areas of all compounds were based on the extracted ion chromatographic peaks. The mean and SD values were calculated and the column diagram of each compound was drawn (Supplementary Table S1). A total of 219 compounds were identified in this study, 73 compounds were not found in RRM, 21 compounds were not found in 4 h PRM, 9 compounds were not found in 8 h PRM and 1 compound was not found in 12 h PRM. During the process of PM, THSG dehydrated with other small molecules to form new compounds.

The change trend of the highest content compound trans-THSG and its cis-THSG slightly increased before 8 h, then decreased gradually, and was lower than that of RRM at 24 h. This is a model of content change, but the peak time may be 4, 8, 12 or 18 h. The second model, such as compound **A3-4** (isomer trans-THSG), gradually increase during the processing time. The third model, such as compound **A45-1** (tetrahydroxystilbene-O-hexoside-feruloyl (glycosyl hydroxyl moiety), gradually decreased with processing time.

The results showed that the content and quantity of stilbene glycoside compounds have undergone tremendous changes during the processing process. Although the content of THSG in PRM is indeed lower than that in RRM, a large number of stilbene glycoside derivatives are produced in the processing process, so the total content of stilbene glycoside compounds in the PRM will not be reduced. Conventional understanding, RRM after processing can enhance efficiency and reduce toxicity, and the content of THSG also decreases with the processing time, is THSG toxic? After this experimental study, it can be proved that THSG should not be a toxic component, because its derivatives will metabolize into compounds similar to THSG *in vivo*, enhancing the efficacy of THSG.

## 4 Conclusion

In the present study, a simple and effective method was developed for characterization of stilbene compounds in the roots of RRM and PRMs by UHPLC-Q-Exactive plus orbitrap MS/MS. Stilbene glycosides were distinguished by diagnostic fragment ions at *m/z* 405.1087 and 243.0656, accurate mass measurements and fragmentation pathways. Based on the proposed strategy, the metabolic process of 7 stilbene glycosides in plants was identified, and 55 substituent and Maillard reaction process were identified. Finally, 219 stilbene glycosides derivatives were identified, of which 102 compounds may be potential new compounds. The 55 substituents include monosaccharide, disaccharide, organic acid and Maillard reaction products (DDMP, 5-HMF and its derivatives) and so on. The quality and quantity of stilbene glycosides changed during the processing of RM. 73 compounds were not found in RRM, 21 compounds were not found in 4 h PRM, 9 compounds were not found in 8 h PRM and 1 compound was not found in 12 h PRM, and the change trend of the compounds can be summarized into 3 models: gradually increased, gradually decreased, first increased and then decreased. 181 trans-THSG derivative products were obtained through the hydrolysis and dehydration reaction between trans-THSG and small molecules compounds, after this experimental study, it can be proved that THSG should not be a toxic component, because its derivatives will metabolize into compounds similar to THSG *in vivo*, enhancing the efficacy of THSG.

## Data Availability

The raw data supporting the conclusion of this article will be made available by the authors, without undue reservation.

## Abbreviations

DDMP, 2, 3-dihydro-3, 5-dihydroxy-6-methyl-4H-pyranone; 5-HMF, 5-hydromethylfurfura; RM, *Reynoutria multiflora* Thunb; PRM, processed *Reynoutria multiflora*; RRM, raw *Reynoutria multiflora*; TCM, Traditional Chinese Medicine; Trans-THSG, tran-2, 3, 5, 4′-tetrahydroxystilbene-2-O-β-D-glucopyranoside; UHPLC-Q-Exactive plus orbitrap MS/MS, ultra high performance liquid chromatography tandem quadrupole/electrostatic field orbitrap high-resolution mass spectrometry.
