# Supplementary material for: Transformation of Stilbene Glucosides From Reynoutria multiflora During Processing
Source: Front Pharmacol. 2022 Apr 25;13:757490. doi: 10.3389/fphar.2022.757490 (PMC9082504; doi:10.3389/fphar.2022.757490)
Supplement: Supplementary file 1 [file Table1.docx]

***Supplementary Material***

Supplementary Table 1 Identification and change curve of the stilbene compounds in RRM and PRM by UPLC-Q-Exactive MS.

| No. | t_R_(min) | [M-H]^-^ | Error (ppm) | Formula | fragment ions | identification | change curve | | Source |
| --- | --- | --- | --- | --- | --- | --- | --- | --- | --- |
| A1-1 | 11.73 | 375.1081 | -1.175 | C_19_H_19_O_8_ | 243.0654(100) | tetrahydroxystilbene-O-pentose ^a^ | 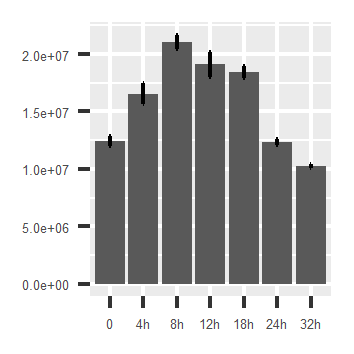 | | RRM, PRM 4h, 8h, 12h, 18h, 24, 32h |
| A1-2 | 12.45 | 375.1081 | -1.175 | C_19_H_19_O_8_ | 243.0654(100) |  | 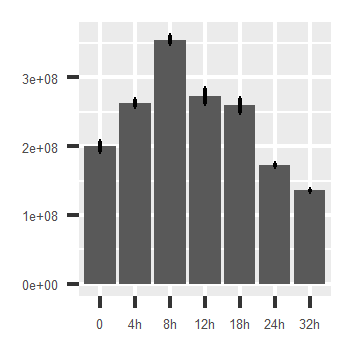 | | RRM, PRM 4h, 8h, 12h, 18h, 24, 32h |
| A2-1 | 14.88 | 389.1242 | 0.024 | C_20_H_21_O_8_ | 243.0659(100%) | tetrahydroxystilbene-O-deoxyhexoside ^a^ | 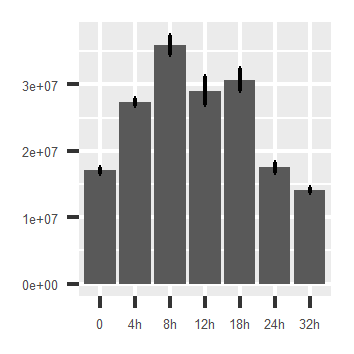 | | RRM, PRM 4h, 8h, 12h, 18h, 24, 32h |
| A2-2 | 15.58 | 389.1243 | 0.281 | C_20_H_21_O_8_ | 243.0659(100%) |  | 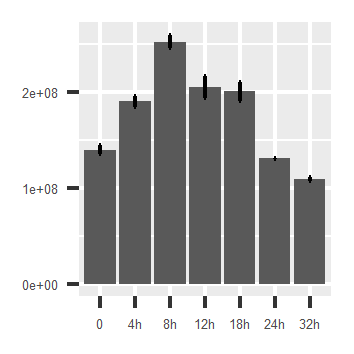 | | RRM, PRM 4h, 8h, 12h, 18h, 24, 32h |
| A2-3 | 16.49 | 389.1242 | 0.024 | C_20_H_21_O_8_ | 243.0659(100%) |  | 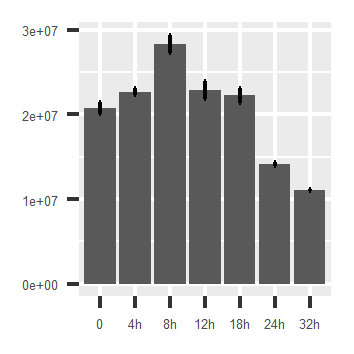 | | RRM, PRM 4h, 8h, 12h, 18h, 24, 32h |
| A3-1 | 6.68 | 405.1187 | -1.001 | C_20_H_21_O_9_ | 243.0655(100%) | cis-THSG isomer ^a^ | 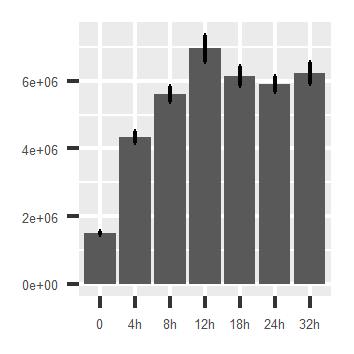 | | RRM, PRM 4h, 8h, 12h, 18h, 24, 32h |
| A3-2 | 7.57 | 405.1187 | -1.001 | C_20_H_21_O_9_ | 243.0656(100%) | cis-THSG ^C^ | 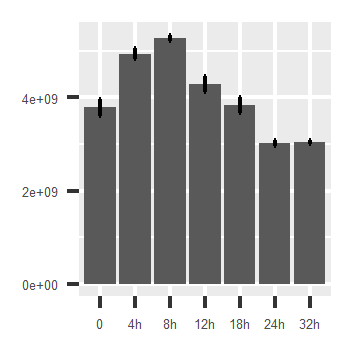 | | RRM, PRM 4h, 8h, 12h, 18h, 24, 32h |
| A3-3 | 9.76 | 405.1187 | -1.001 | C_20_H_21_O_9_ | 243.0654(100%) | trans-astringin^c^ | 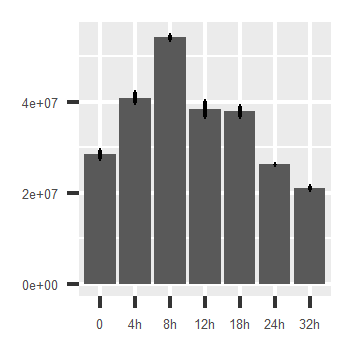 | | RRM, PRM 4h, 8h, 12h, 18h, 24, 32h |
| A3-4 | 10.26 | 405.1187 | -1.001 | C_20_H_21_O_9_ | 243.0654(100%) | trans-THSG isomer ^a^ | 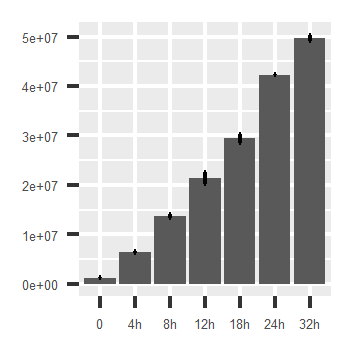 | | RRM, PRM 4h, 8h, 12h, 18h, 24, 32h |
| A3-5 | 11.28 | 405.1191 | -0.013 | C_20_H_21_O_9_ | 243.0655(100%) | trans-THSG ^C^ | 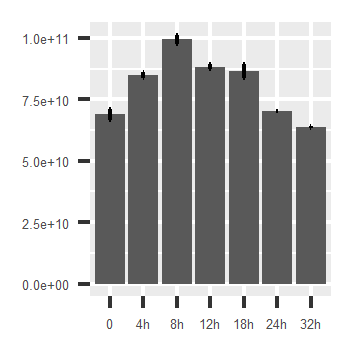 | | RRM, PRM 4h, 8h, 12h, 18h, 24, 32h |
| A4-1 | 4.08 | 423.1295 | -0.402 | C_20_H_23_O_10_ | 261.0764(100%) 243.0654(25%) | unkown | 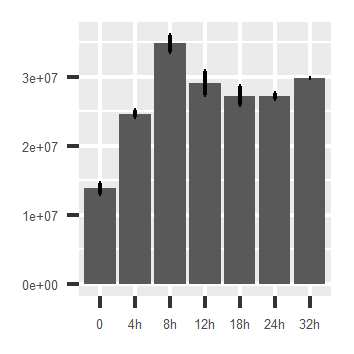 | | RRM, PRM 4h, 8h, 12h, 18h, 24, 32h |
| A4-2 | 4.42 | 423.1294 | -0.638 | C_20_H_23_O_10_ | 261.0764(100%) 243.0654(25%) |  | 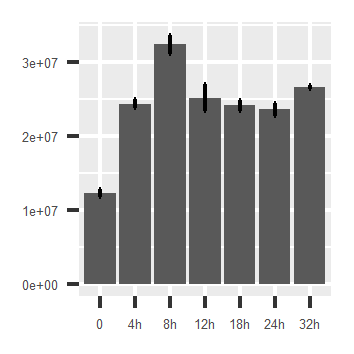 | | RRM, PRM 4h, 8h, 12h, 18h, 24, 32h |
| A5-1 | 13.63 | 433.1136 | -0.970 | C_21_H_21_O_10_ | 271.0608(100%) | tetrahydroxystilbene-O-hexoside-O-formic acid acyl (phenolic hydroxyl moiety) ^b^ | 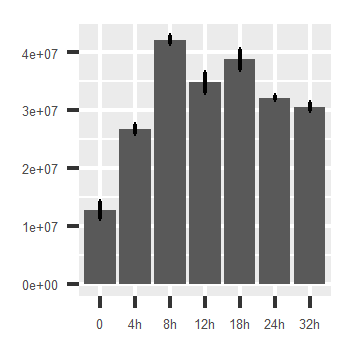 | | RRM, PRM 4h, 8h, 12h, 18h, 24, 32h |
| A5-2 | 14.95 | 433.1136 | -0.970 | C_21_H_21_O_10_ | 271.0608(10%) 243.0654(100%） |  | 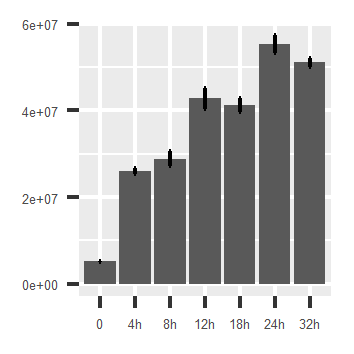 | | RRM, PRM 4h, 8h, 12h, 18h, 24, 32h |
| A5-3 | 19.78 | 433.1139 | -0.277 | C_21_H_21_O_10_ | 271.0609(100%) |  | 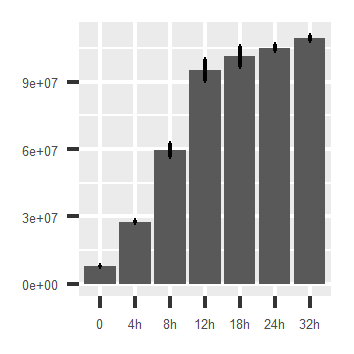 | | RRM, PRM 4h, 8h, 12h, 18h, 24, 32h |
| A5-4 | 22.31 | 433.1140 | -0.046 | C_21_H_21_O_10_ | 271.0609(100%) 243.0652(10%) |  | 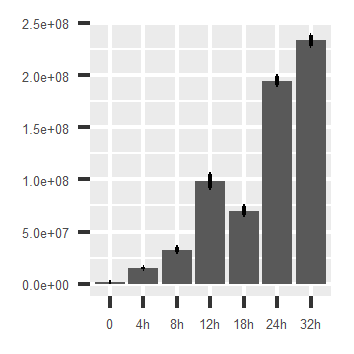 | | RRM, PRM 4h, 8h, 12h, 18h, 24, 32h |
| A6-1 | 7.15 | 437.1450 | -0.732 | C_21_H_25_O_10_ | 243.0655(100%) | unkown ^b^ | 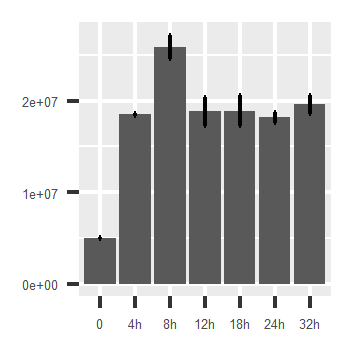 | | RRM, PRM 4h, 8h, 12h, 18h, 24, 32h |
| A6-2 | 7.37 | 437.1448 | -1.19 | C_21_H_25_O_10_ | 243.0655(100%) |  | 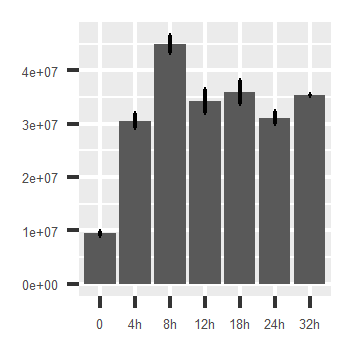 | | RRM, PRM 4h, 8h, 12h, 18h, 24, 32h |
| A7-1 | 14.46 | 447.1298 | 0.291 | C_22_H_23_O_10_ | 243.0655(100%) | tetrahydroxystilbene-O-(acetyl)-hexoside ^a^ | 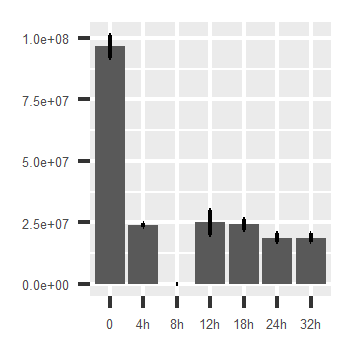 | | RRM, PRM 4h, 12h, 18h, 24, 32h |
| A7-2 | 15.28 | 447.1300 | 0.738 | C_22_H_23_O_10_ | 243.0655(100%) |  | 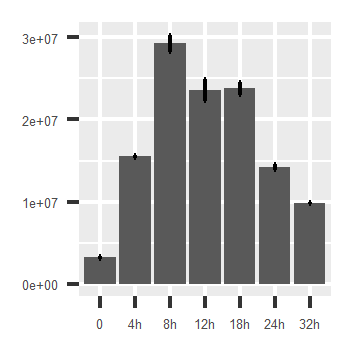 | | RRM, PRM 4h, 8h, 12h, 18h, 24, 32h |
| A7-3 | 15.63 | 447.1294 | -0.604 | C_22_H_23_O_10_ | 243.0655(100%) |  | 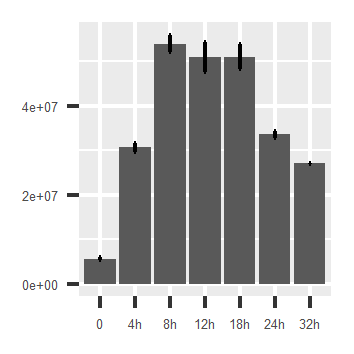 | | RRM, PRM 4h, 8h, 12h, 18h, 24, 32h |
| A7-4 | 16.1 | 447.1293 | -0.828 | C_22_H_23_O_10_ | 243.0655(100%) |  | 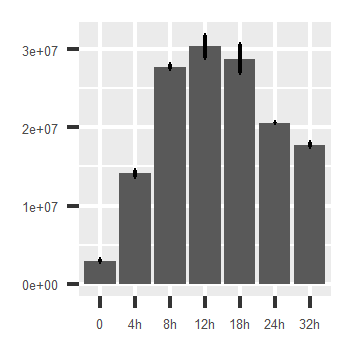 | | RRM, PRM 4h, 8h, 12h, 18h, 24, 32h |
| A7-5 | 16.5 | 447.1299 | 0.514 | C_22_H_23_O_10_ | 243.0655(100%) |  | 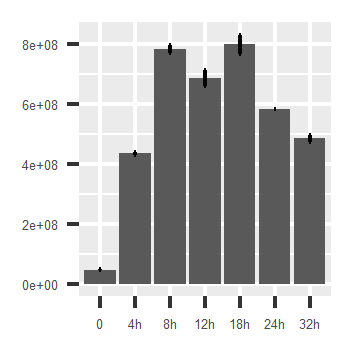 | | RRM, PRM 4h, 8h, 12h, 18h, 24, 32h |
| A8-1 | 12.48 | 449.1086 | -0.745 | C_21_H_21_O_11_ | 287.0554(100%) 243.0655(30%) | tetrahydroxystilbene-O-hexoside-O-carbonate acyl (phenolic hydroxyl moiety) ^b^ | 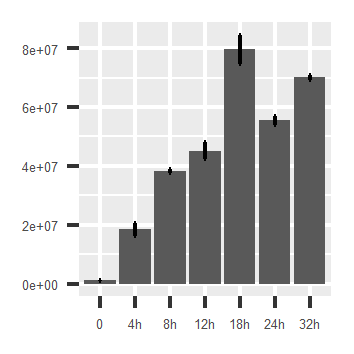 | | RRM, PRM 4h, 8h, 12h, 18h, 24, 32h |
| A8-2 | 14.35 | 449.1089 | -0.077 | C_21_H_21_O_11_ | 287.0554(100%) 243.0655(30%) |  | 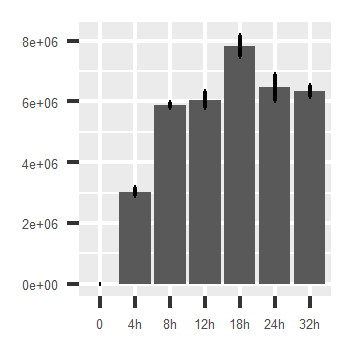 | | PRM 4h, 8h, 12h, 18h, 24, 32h |
| A9 | 15.67 | 457.1116 | 1.691 | C_23_H_21_O_10_ | 243.0654(100%) | tetrahydroxystilbene-O-(hydroxycyclopropenon)-hexoside ^b^ | 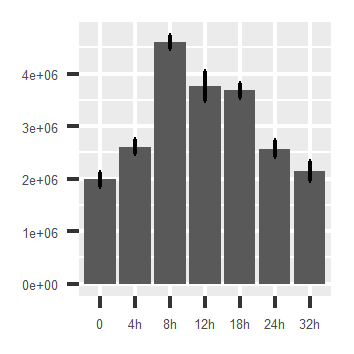 | | RRM, PRM 4h, 8h, 12h, 18h, 24, 32h |
| A10 | 16.77 | 459.1289 | -1.677 | C_23_H_23_O_10_ | 243.0656(100%) | tetrahydroxystilbene-O- (acrylic acid acyl)-hexoside ^b^ | 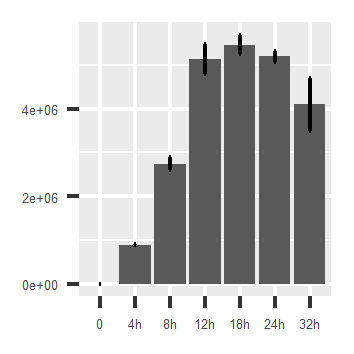 | | PRM 4h, 8h, 12h, 18h, 24, 32h |
| A11 | 14.86 | 461.1449 | -0.911 | C_23_H_25_O_10_ | 299.0910(23%) 243.0655(100%) | tetrahydroxystilbene-O- (propionic acid acyl)-hexoside ^b^ | 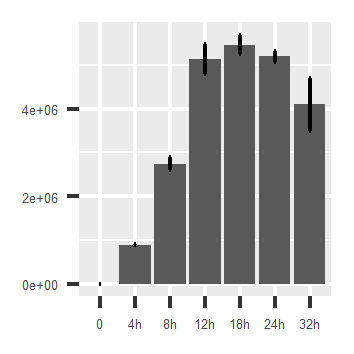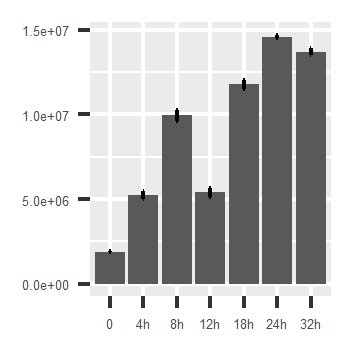 | | RRM, PRM 4h, 8h, 12h, 18h, 24, 32h |
| A12-1 | 11.76 | 463.1247 | 0.249 | C_22_H_23_O_11_ | 243.0654(40%) | tetrahydroxystilbene-O- (glycolic acid acyl)-hexoside ^b^ | 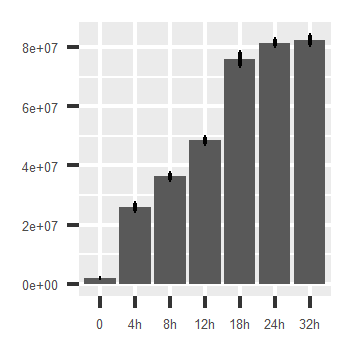 | | RRM, PRM 4h, 8h, 12h, 18h, 24, 32h |
| A12-2 | 13.37 | 463.1244 | -0.399 | C_22_H_23_O_11_ | 243.0654(100%) |  | 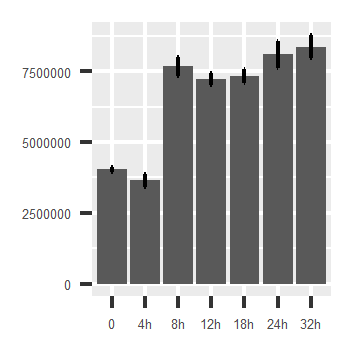 | | RRM, PRM 4h, 8h, 12h, 18h, 24, 32h |
| A13-1 | 7.8 | 477.1393 | -1.959 | C_23_H_25_O_11_ | 405.1184(10%) 315.0859(35%) 297.0756(20%) 243.0653(100%) | tetrahydroxystilbene-O-hexoside-O-lactic acid acyl (phenolic hydroxyl moiety) ^b^ | 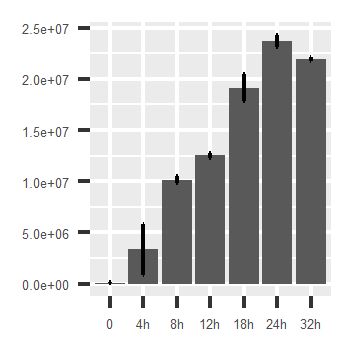 | | RRM, PRM 4h, 8h, 12h, 18h, 24, 32h |
| A13-2 | 10.64 | 477.1396 | -1.330 | C_23_H_25_O_11_ | 405.1184(10%) 315.0859(90%) 297.0756(30%) 243.0653(100%) |  | 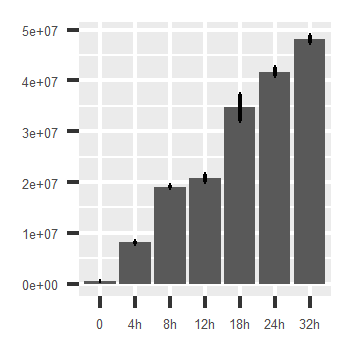 | | RRM, PRM 4h, 8h, 12h, 18h, 24, 32h |
| A13-3 | 10.85 | 477.1396 | -1.330 | C_23_H_25_O_11_ | 405.1184(10%) 315.0859(90%) 297.0756(30%) 243.0653(100%) |  | 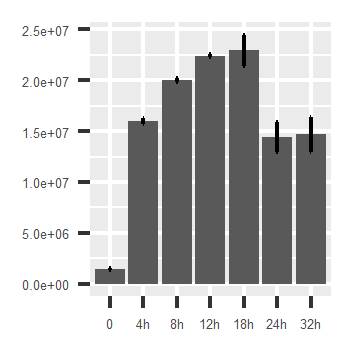 | | RRM, PRM 4h, 8h, 12h, 18h, 24, 32h |
| A14-1 | 20.76 | 489.1758 | -1.677 | C_25_H_29_O_10_ | 405.1176(7%) 327.1222(10%) 243.0656(100%) | tetrahydroxystilbene-O-hexoside-O-valerate acyl (phenolic hydroxyl moiety) ^b^ | 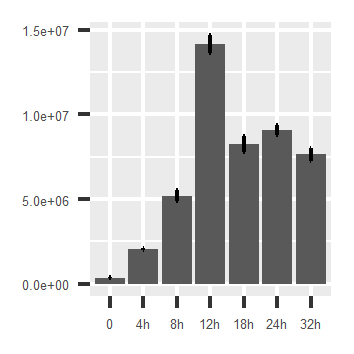 | | RRM, PRM 4h, 8h, 12h, 18h, 24, 32h |
| A14-2 | 20.99 | 489.1759 | -1.472 | C_25_H_29_O_10_ | 405.1176(7%) 327.1222(10%) 243.0656(100%) |  | 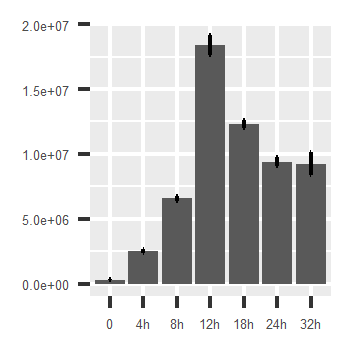 | | RRM, PRM 4h, 8h, 12h, 18h, 24, 32h |
| A14-3 | 22.36 | 489.1761 | -1.064 | C_25_H_29_O_10_ | 405.1176(7%) 327.1222(10%) 243.0656(100%) |  | 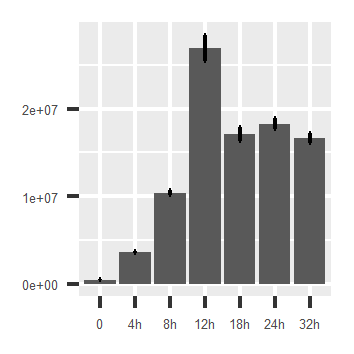 | | RRM, PRM 4h, 8h, 12h, 18h, 24, 32h |
| A14-4 | 22.81 | 489.1763 | -0.655 | C_25_H_29_O_10_ | 405.1176(7%) 327.1222(10%) 243.0656(100%) |  | 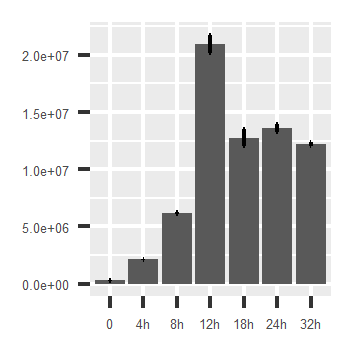 | | RRM, PRM 4h, 8h, 12h, 18h, 24, 32h |
| A15 | 19.99 | 499.1241 | -0.971 | C_25_H_23_O_11_ | 337.0704(100%) 293.0812(55%) 243.0656(45%) | tetrahydroxystilbene-O-hexoside-O-5-hydroxyfuran-2-carbaldehyde (phenolic hydroxyl moiety) ^b^ | 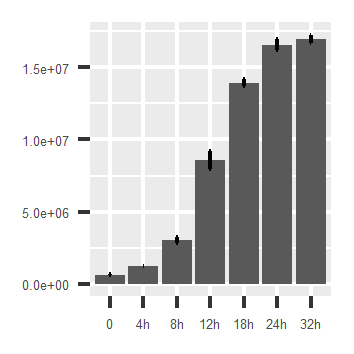 | | RRM, PRM 4h, 8h, 12h, 18h, 24, 32h |
| A16-1 | 8.85 | 501.1396 | -1.267 | C_25_H_25_O_11_ | 339.0858(100%) 321.0755(15%) 295.0968(15%) 243.0653(25%) | tetrahydroxystilbene-O-hexoside-O-4-hydroxymethyl-5H-furan-2-one (phenolic hydroxyl moiety) ^b^ | 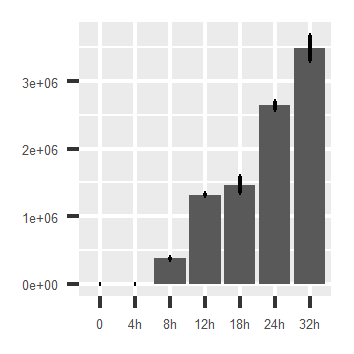 | | PRM 8h, 12h, 18h, 24, 32h |
| A16-2 | 10.01 | 501.1393 | -1.865 | C_25_H_25_O_11_ | 339.0859(65%) 321.0756(40%) 243.0654(100%) |  | 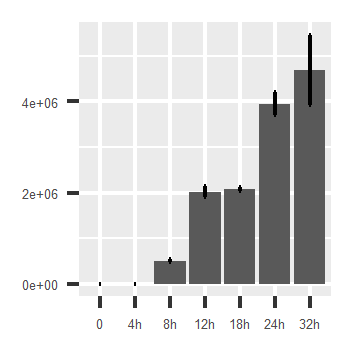 | | PRM 8h, 12h, 18h, 24, 32h |
| A17 | 18.06 | 503.1553 | -1.162 | C_25_H_27_O_11_ | 341.1019(65%) 297.1125(100%) 243.0654(25%) | tetrahydroxystilbene-O-hexoside-O-5-hydroxymethyl-4,5-dihydrofuranone (phenolic hydroxyl moiety) ^b^ | 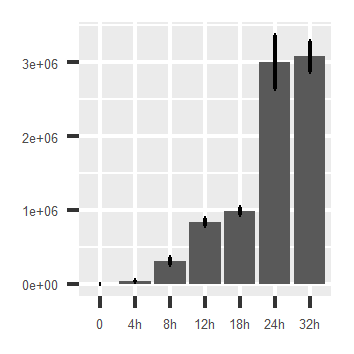 | | PRM 4h, 8h, 12h, 18h, 24, 32h |
| A18-1 | 9.53 | 505.1346 | -1.088 | C_24_H_25_O_12_ | 405.1178(23%) 243.0655(100%) | tetrahydroxystilbene-O- (succinic acid acyl)-hexoside ^b^ | 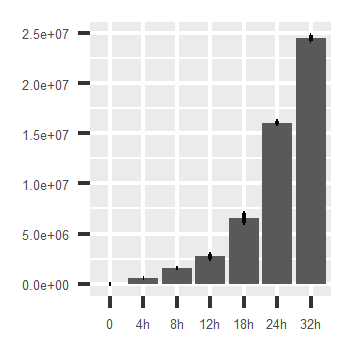 | | PRM 4h, 8h, 12h, 18h, 24, 32h |
| A18-2 | 10.21 | 505.1343 | -1.681 | C_24_H_25_O_12_ | 405.1174(20%) 243.0655(100%) |  | 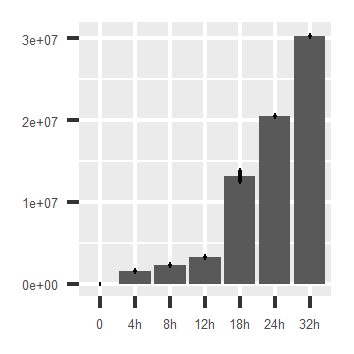 | | PRM 4h, 8h, 12h, 18h, 24, 32h |
| A18-3 | 13.31 | 505.1346 | -1.088 | C_24_H_25_O_12_ | 343.0813(100%) 243.0655(20%) | tetrahydroxystilbene-O-hexoside-O-succinic acid acyl (phenolic hydroxyl moiety) ^b^ | 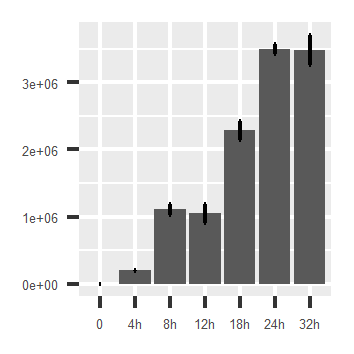 | | PRM 4h, 8h, 12h, 18h, 24, 32h |
| A18-4 | 15.61 | 505.1347 | -0.890 | C_24_H_25_O_12_ | 343.0799(10%) 243.0655(100%) |  | 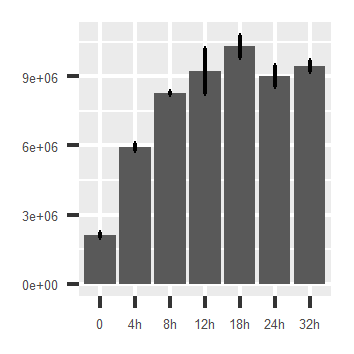 | | RRM, PRM 4h, 8h, 12h, 18h, 24, 32h |
| A18-5 | 16.01 | 505.1353 | 1.247 | C_24_H_25_O_12_ | 343.0812(90%) 243.0655(100%) |  | 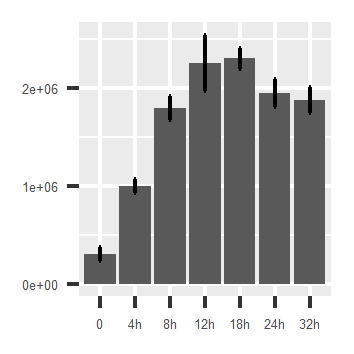 | | RRM, PRM 4h, 8h, 12h, 18h, 24, 32h |
| A19-1 | 11.85 | 507.1500 | -1.576 | C_24_H_27_O_12_ | 345.0966(40%) 313.0709(20%) 285.0763(10%) 255.0656(40%) 243.0655(100%) | tetrahydroxystilbene-O-hexoside-O-dihydroxybutyrate (phenolic hydroxyl moiety) ^b^ | 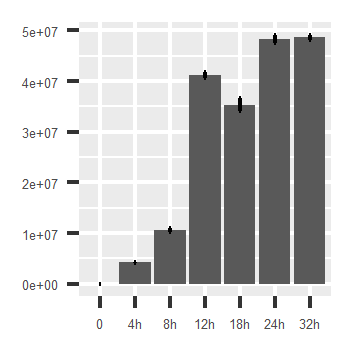 | | PRM 4h, 8h, 12h, 18h, 24, 32h |
| A19-2 | 12 | 507.1500 | -1.576 | C_24_H_27_O_12_ | 345.0966(40%) 313.0709(20%) 285.0763(10%) 255.0656(40%) 243.0655(100%) |  | 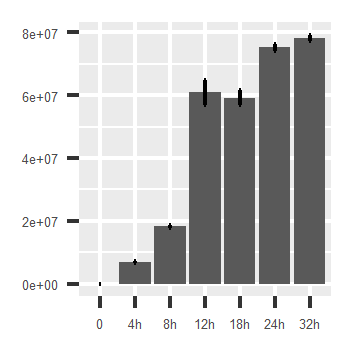 | | PRM 4h, 8h, 12h, 18h, 24, 32h |
| A20 | 18.4 | 511.1603 | -1.311 | C_27_H_27_O_10_ | 349.1068(100%) 243.0655(50%) | tetrahydroxystilbene-O-hexoside-O-salicyloyl (phenolic hydroxyl moiety) ^a^ | 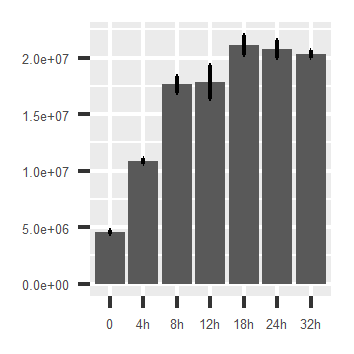 | | RRM, PRM 4h, 8h, 12h, 18h, 24, 32h |
| A21-1 | 17.99 | 512.1555 | -1.404 | C_26_H_26_O_10_N | 405.1175(10%)243.0655(100%) | tetrahydroxystilbene-O-(aminocatecholoyl)-hexosides ^b^ | 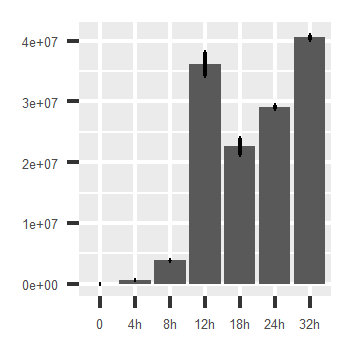 | | PRM 4h, 8h, 12h, 18h, 24, 32h |
| A21-2 | 19.42 | 512.1558 | -0.818 | C_26_H_26_O_10_N | 405.1180(10%)349.0979(100%)243.0655(85%) |  | 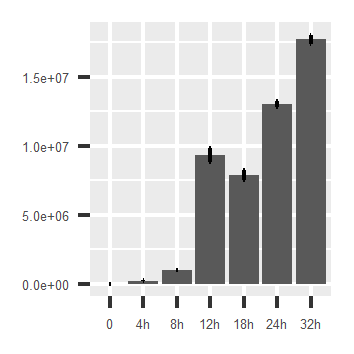 | | PRM 4h, 8h, 12h, 18h, 24, 32h |
| A22-1 | 14.67 | 513.1397 | -1.042 | C_26_H_25_O_11_ | 351.0862(100%) 243.0654(20%) | tetrahydroxystilbene-O-hexoside-O-5-HMF (phenolic hydroxyl moiety) ^b^ | 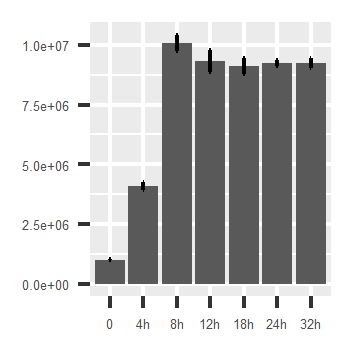 | | RRM, PRM 4h, 8h, 12h, 18h, 24, 32h |
| A22-2 | 16.44 | 513.1394 | -1.627 | C_26_H_25_O_11_ | 351.0857(100%) 243.0654(15%) |  | 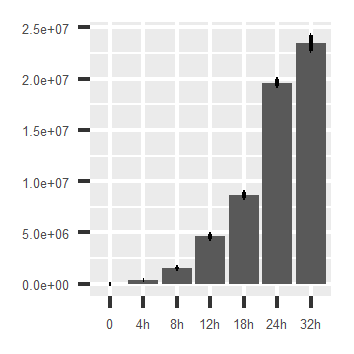 | | PRM 4h, 8h, 12h, 18h, 24, 32h |
| A22-3 | 18.99 | 513.1397 | -1.042 | C_26_H_25_O_11_ | 351.0859(100%) 243.0655(10%） |  | 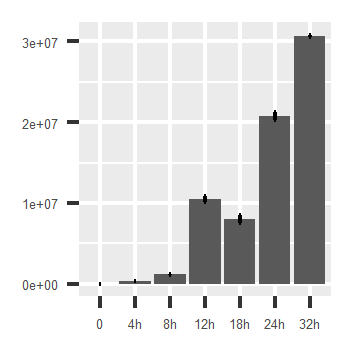 | | PRM 4h, 8h, 12h, 18h, 24, 32h |
| A22-4 | 21.16 | 513.1392 | -2.017 | C_26_H_25_O_11_ | 243.0655(100%) | tetrahydroxystilbene-O-(5-HMF)-hexoside ^b^ | | 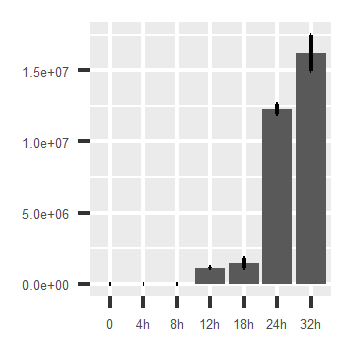 | PRM 12h, 18h, 24, 32h |
| A23-1 | 15.29 | 515.1555 | -0.747 | C_26_H_27_O_11_ | 353.1021(100%) 243.0654(25%) | tetrahydroxystilbene-O-hexoside-O-2,5-bis-(hydroxymethyl)furan (phenolic hydroxyl moiety) ^b^ | 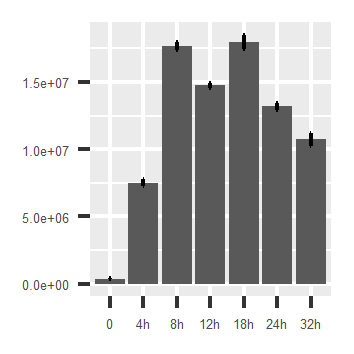 | | RRM, PRM 4h, 8h, 12h, 18h, 24, 32h |
| A23-2 | 15.69 | 515.1555 | -0.747 | C_26_H_27_O_11_ | 405.1173(18%) 353.1021(90%) 243.0654(100%) |  | 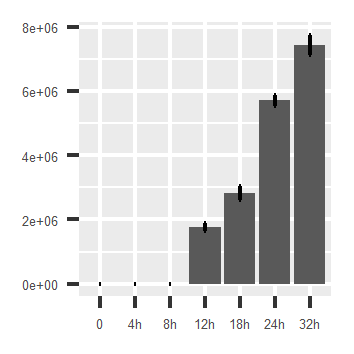 | | PRM 12h, 18h, 24, 32h |
| A24 | 16.49 | 515.1179 | -3.105 | C_25_H_23_O_12_ | 243.0656(100%) | tetrahydroxystilbene-O-(5-hydroxyfuran-2-carboxylic acid)-hexoside ^b^ | 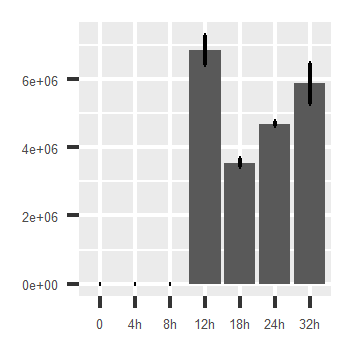 | | PRM 12h, 18h, 24, 32h |
| A25 | 14.27 | 516.1503 | -1.615 | C_25_H_26_O_11_N | 243.0655(100%)128.0336(85%) | tetrahydroxystilbene-O-(pyroglutamyl)-hexoside ^b^ | 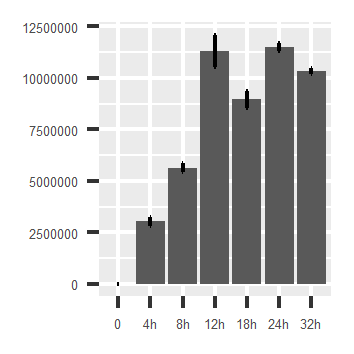 | | PRM 4h, 8h, 12h, 18h, 24, 32h |
| A26-1 | 7.92 | 519.1499 | -1.732 | C_25_H_27_O_12_ | 405.1167(10%) 357.0967(100%) 339.0865(20%) 297.0760(55%) 243.0655(20%) | tetrahydroxystilbene-O-(glutaryl)-hexoside ^b^ | | 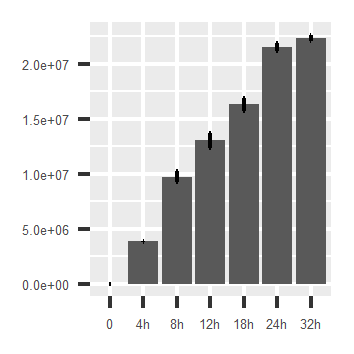 | PRM 4h, 8h, 12h, 18h, 24, 32h |
| A26-2 | 8.33 | 519.1495 | -2.503 | C_25_H_27_O_12_ | 357.0967(100%) 339.0858(80%)297.0760(90%)243.0655(20%) | tetrahydroxystilbene-O-hexoside-O-glutaryl (phenolic hydroxyl moiety) ^b^ | 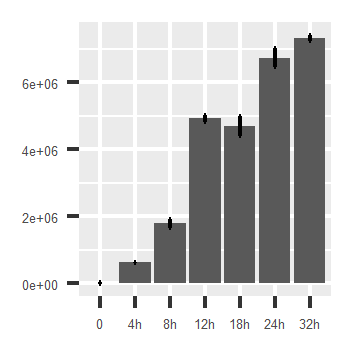 | | RRM, PRM 4h, 8h, 12h, 18h, 24, 32h |
| A26-3 | 13.86 | 519.1503 | -0.962 | C_25_H_27_O_12_ | 405.1167(10%) 357.0966(100%) 339.0855(20%) 297.0760(55%) 243.0655(20%) |  | 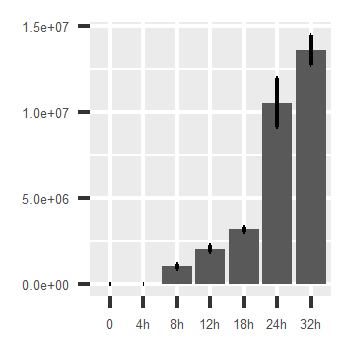 | | PRM 8h, 12h, 18h, 24, 32h |
| A26-4 | 17.54 | 519.1503 | -0.962 | C_25_H_27_O_12_ | 243.0655(100%) |  | 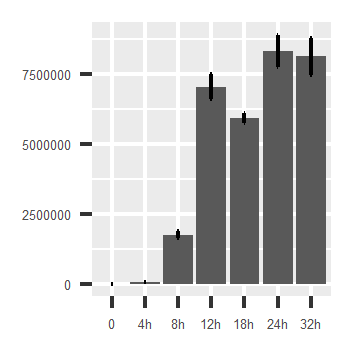 | | PRM 4h, 8h, 12h, 18h, 24, 32h |
| A27-1 | 12.84 | 521.1294 | -1.274 | C_24_H_25_O_13_ | 405.1183(10%) 243.0657（100%） | tetrahydroxystilbene-O-hexoside-O-malic acid acyl (phenolic hydroxyl moiety) ^a^ | 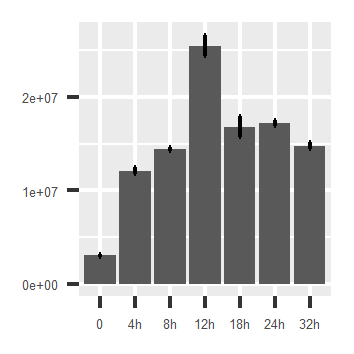 | | RRM, PRM 4h, 8h, 12h, 18h, 24, 32h |
| A27-2 | 13.06 | 521.1292 | -1.658 | C_24_H_25_O_13_ | 405.1177(10%) 243.0657（100%） |  | 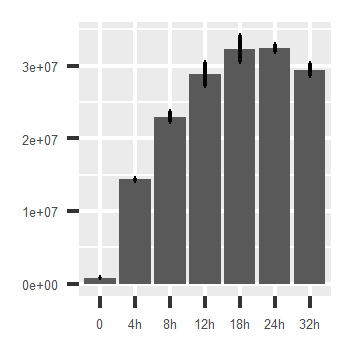 | | RRM, PRM 4h, 8h, 12h, 18h, 24, 32h |
| A27-3 | 13.82 | 521.1293 | -1.466 | C_24_H_25_O_13_ | 405.1175(10%) 359.1115(10%) 243.0654(100%) |  | 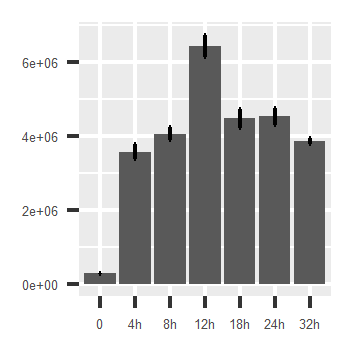 | | RRM, PRM 4h, 8h, 12h, 18h, 24, 32h |
| A28-1 | 17.04 | 525.1396 | -1.209 | C_27_H_25_O_11_ | 525.1396(23%) 363.0855(100%) 243.0656(8%) | tetrahydroxystilbene-O-hexoside-O-salicylic acid acyl (phenolic hydroxyl moiety) ^a^ | 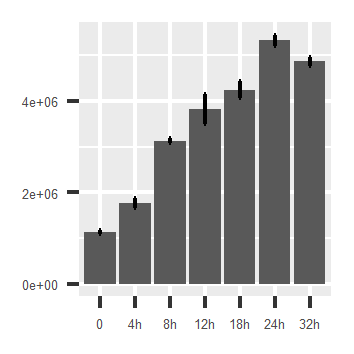 | | RRM, PRM 4h, 8h, 12h, 18h, 24, 32h |
| A28-2 | 18.26 | 525.1396 | -1.209 | C_27_H_25_O_11_ | 405.1179(10%) 363.0863(23%) 243.0656(100%) 137.0228(38%) |  | 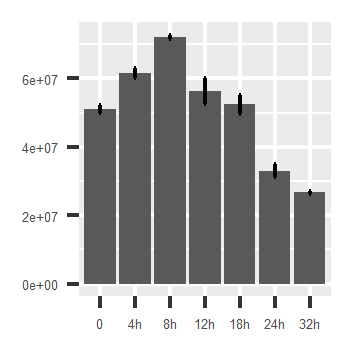 | | RRM, PRM 4h, 8h, 12h, 18h, 24, 32h |
| A28-3 | 19.21 | 525.1396 | -1.209 | C_27_H_25_O_11_ | 405.1179(10%) 363.0863(10%) 243.0656(100%) 137.0228(28%) |  | 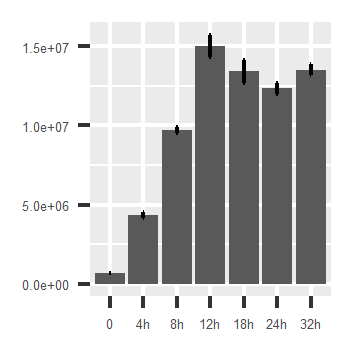 | | RRM, PRM 4h, 8h, 12h, 18h, 24, 32h |
| A28-4 | 21.24 | 525.1390 | -2.351 | C_27_H_25_O_11_ | 405.1179(10%) 243.0656(100%) 137.0228(44%) | tetrahydroxystilbene-O- (salicylic acid acyl)-hexosides ^a^ | 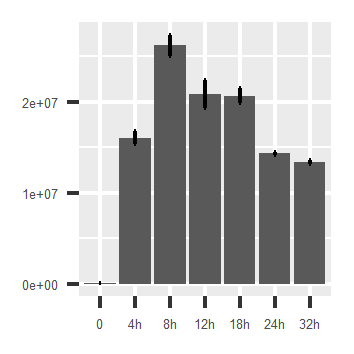 | | RRM, PRM 4h, 8h, 12h, 18h, 24, 32h |
| A28-5 | 25.12 | 525.1396 | -1.209 | C_27_H_25_O_11_ | 243.0657(30%) 137.0229(100%) |  | 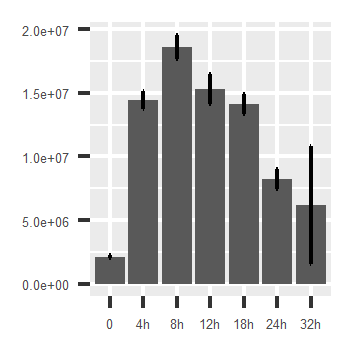 | | RRM, PRM 4h, 8h, 12h, 18h, 24, 32h |
| A29-1 | 15.65 | 527.1190 | -0.947 | C_26_H_23_O_12_ | 365.0652(100%) 243.0658(20%） | tetrahydroxystilbene-O-5-formylfuran-2-carboxylyl-hexosides (phenolic hydroxyl moiety) ^b^ | 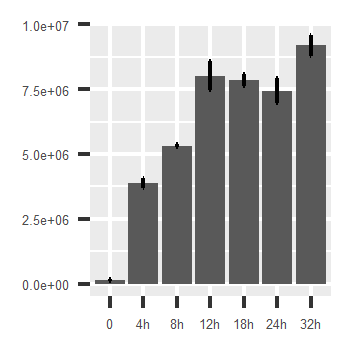 | | RRM, PRM 4h, 8h, 12h, 18h, 24, 32h |
| A29-2 | 18.37 | 527.1190 | -0.947 | C_26_H_23_O_12_ | 365.0652(100%) 243.0658(20%） |  | 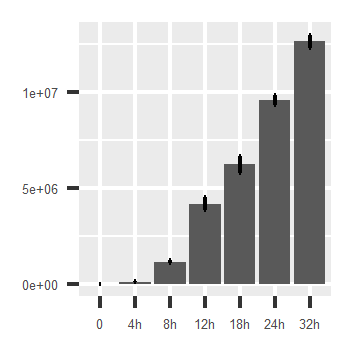 | | PRM 4h, 8h, 12h, 18h, 24, 32h |
| A29-3 | 22.85 | 527.1190 | -0.947 | C_26_H_23_O_12_ | 365.0652(100%) 243.0658(20%） |  | 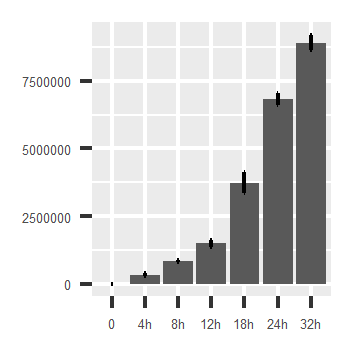 | | PRM 4h, 8h, 12h, 18h, 24, 32h |
| A30-1 | 12.06 | 529.1345 | -1.227 | C_26_H_25_O_12_ | 367.0807(75%) 323.0914(100%) 243.0656(5%) | tetrahydroxystilbene (phenolic hydroxyl moiety)-O-5-hydroxymethylfuran-2-carboxylyl-hexosides (hydroxyl) ^b^ | 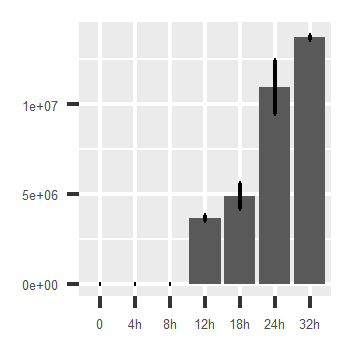 | | PRM 12h, 18h, 24, 32h |
| A30-2 | 13.07 | 529.1343 | -1.605 | C_26_H_25_O_12_ | 367.0807(40%) 323.0914(100%) 243.0656(5%) |  | 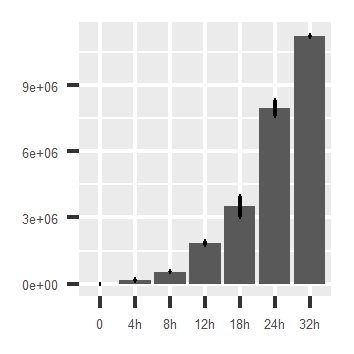 | | PRM 4h, 8h, 12h, 18h, 24, 32h |
| A30-6 | 16.5 | 529.1343 | -1.605 | C_26_H_25_O_12_ | 367.0793(45%)323.0911(100%) 243.0656(40%) |  | 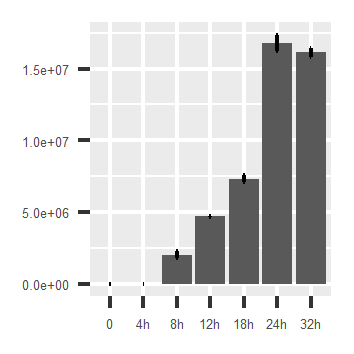 | | PRM 8h, 12h, 18h, 24, 32h |
| A30-3 | 14.31 | 529.1345 | -1.227 | C_26_H_25_O_12_ | 366.0725(32%) 367.0807(30%) 243.0656(100%) 123.0071(72%) | tetrahydroxystilbene (phenolic hydroxyl moiety)-O-5-hydroxymethylfuran-2-carboxylyl-hexoside (carboxyl moiety) ^b^ | 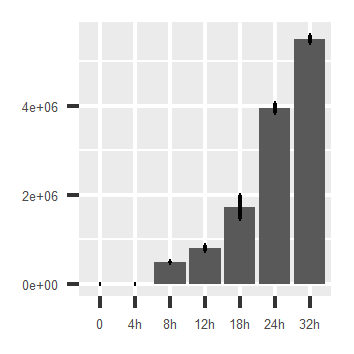 | | PRM 8h, 12h, 18h, 24, 32h |
| A30-4 | 15.3 | 529.1349 | -0.471 | C_26_H_25_O_12_ | 367.0807(100%) 243.0654(30%) |  | 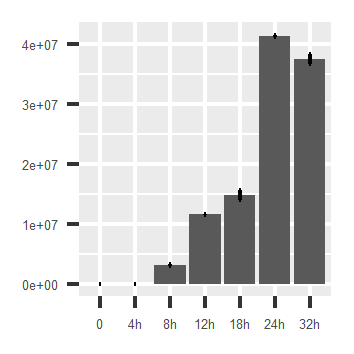 | | PRM 8h, 12h, 18h, 24, 32h |
| A30-5 | 16.14 | 529.1346 | -1.038 | C_26_H_25_O_12_ | 405.1176 (10%) 243.0654(100%) 123.0071(70%) | tetrahydroxystilbene-O-(5-hydroxymethylfuran-2-carboxylyl)-hexoside ^b^ | 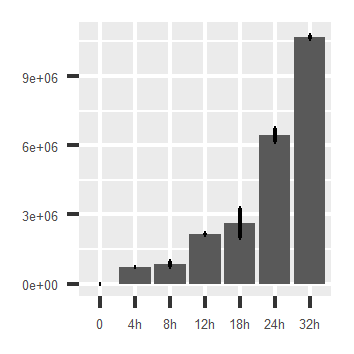 | | PRM 4h, 8h, 12h, 18h, 24, 32h |
| A30-7 | 16.87 | 529.1345 | -1.227 | C_26_H_25_O_12_ | 405.1176 (10%) 243.0654(100%) 123.0071(70%) |  | 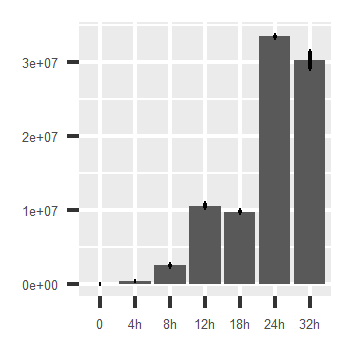 | | PRM 4h, 8h, 12h, 18h, 24, 32h |
| A31-1 | 2.58 | 531.1509 | 0.189 | C_26_H_27_O_12_ | 405.1182(15%) 369.0971(20%) 351.0863(15%) 295.0609(18%) 243.0657(100%) | tetrahydroxystilbene-O-hexoside-DDMP (phenolic hydroxyl moiety) ^b^ | 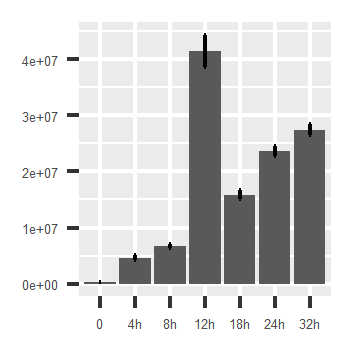 | | RRM, PRM 4h, 8h, 12h, 18h, 24, 32h |
| A31-2 | 2.75 | 531.1505 | -0.564 | C_26_H_27_O_12_ | 405.1179(10%) 369.0970(20%) 295.0609(18%) 243.0657(100%) |  | 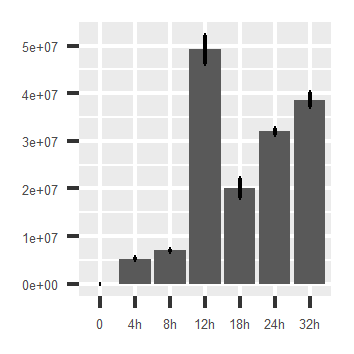 | | PRM 4h, 8h, 12h, 18h, 24, 32h |
| A31-3 | 3.58 | 531.1507 | -0.187 | C_26_H_27_O_12_ | 531.1498(20%) 369.0970(100%) 351.0857(30%) 243.0656(70%) |  | 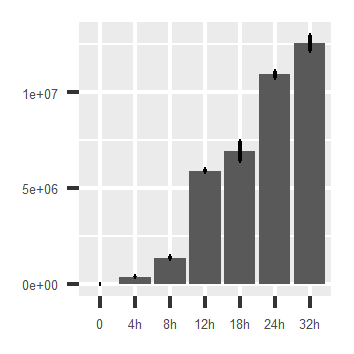 | | PRM 4h, 8h, 12h, 18h, 24, 32h |
| A31-4 | 4.66 | 531.1508 | 0.001 | C_26_H_27_O_12_ | 369.0970(100%) 351.0858(18%) 243.0657(38%) |  | 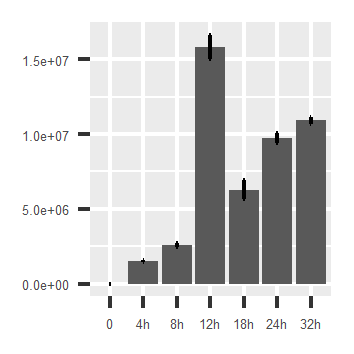 | | PRM 4h, 8h, 12h, 18h, 24, 32h |
| A31-5 | 5 | 531.1507 | -0.187 | C_26_H_27_O_12_ | 369.0971(100%) 243.0657(30%) |  | 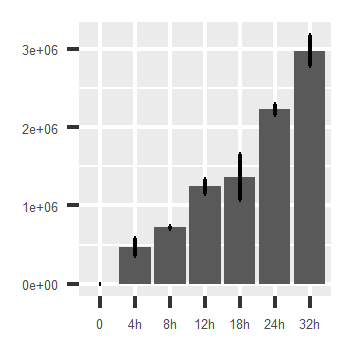 | | PRM 4h, 8h, 12h, 18h, 24, 32h |
| A31-6 | 5.64 | 531.1508 | 0.001 | C_26_H_27_O_12_ | 369.0971(100%) 297.0765(10%) 243.0655 (10%) |  | 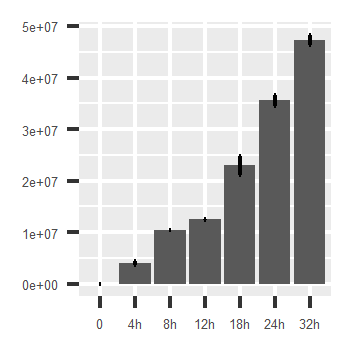 | | PRM 4h, 8h, 12h, 18h, 24, 32h |
| A31-7 | 12.14 | 531.1511 | 0.566 | C_26_H_27_O_12_ | 405.1179(28%) 369.0958(21%) 351.0850(10%) 295.0611(10%) 243.0655(100%) |  | 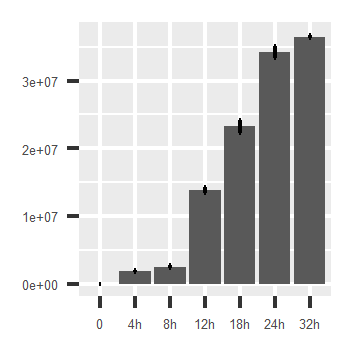 | | PRM 4h, 8h, 12h, 18h, 24, 32h |
| A31-8 | 15.02 | 531.1507 | -0.187 | C_26_H_27_O_12_ | 405.1180(20%) 369.0966(22%) 295.0603(43%) 243.0657(100%) |  | 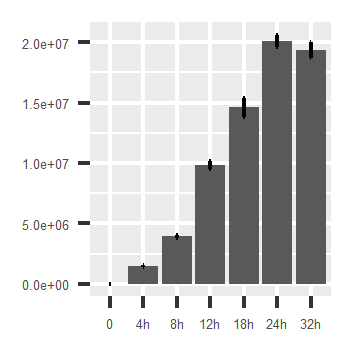 | | PRM 4h, 8h, 12h, 18h, 24, 32h |
| A31-9 | 15.36 | 531.1506 | -0.375 | C_26_H_27_O_12_ | 405.1179(10%) 369.0967100%) 351.0862(50%) 243.0655(50%) |  | 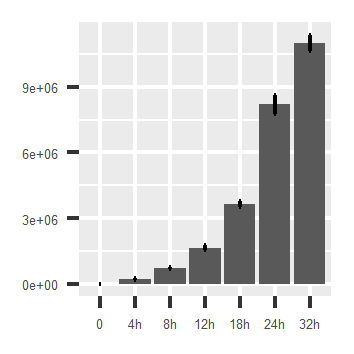 | | PRM 4h, 8h, 12h, 18h, 24, 32h |
| A31-10 | 15.74 | 531.1505 | -0.564 | C_26_H_27_O_12_ | 405.1184(10%) 369.0966100%) 351.0862(60%) 243.0655(24%) |  | 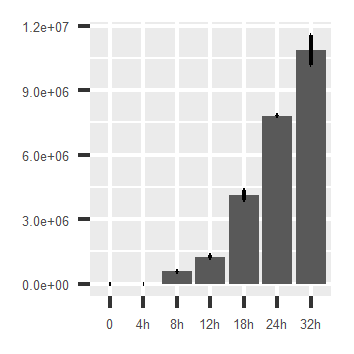 | | PRM 8h, 12h, 18h, 24, 32h |
| A32-1 | 7.97 | 533.1658 | -1.218 | C_26_H_29_O_12_ | 371.1124(100%) 327.0863(12%) 243.0657(10%) | tetrahydroxystilbene-O-hexoside-adipic acid acyl (phenolic hydroxyl moiety) ^b^ | 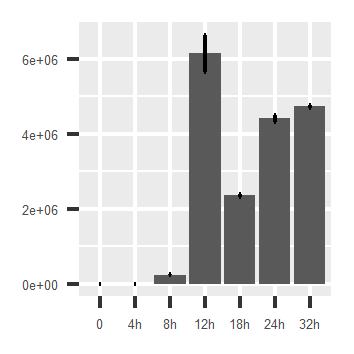 | | PRM 8h, 12h, 18h, 24, 32h |
| A32-2 | 10.19 | 533.1658 | -1.218 | C_26_H_29_O_12_ | 371.1124(100%) 327.0863(12%) 243.0657(10%) |  | 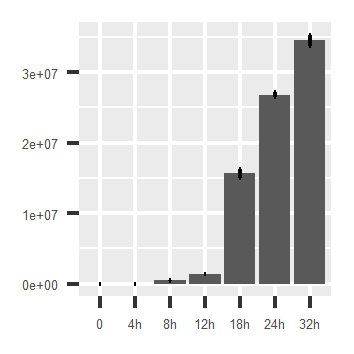 | | PRM 8h, 12h, 18h, 24, 32h |
| A32-3 | 10.64 | 533.1656 | -1.593 | C_26_H_29_O_12_ | 371.1124(100%) 327.0863(12%) 243.0657(10%) |  | 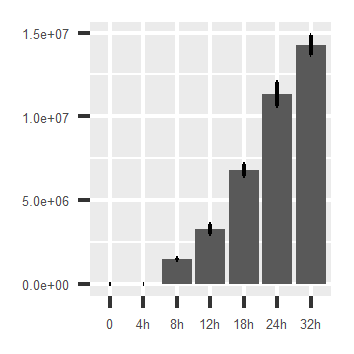 | | PRM 8h, 12h, 18h, 24, 32h |
| A32-4 | 12.18 | 533.1660 | -0.843 | C_26_H_29_O_12_ | 371.1122(100%) 243.0657(10%) |  | 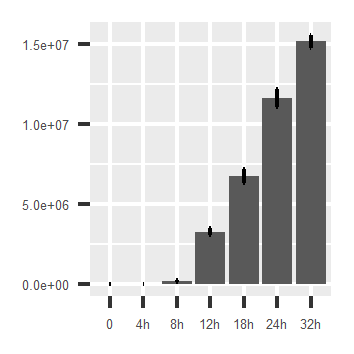 | | PRM 8h, 12h, 18h, 24, 32h |
| A32-5 | 12.98 | 533.1657 | -1.406 | C_26_H_29_O_12_ | 371.1124(100%) 327.0863(12%) 243.0657(10%) |  | 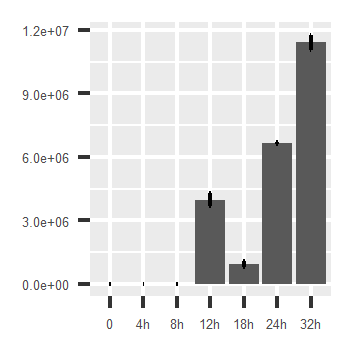 | | PRM 12h, 18h, 24, 32h |
| A33-1 | 12.1 | 537.1605 | 0.233 | C_25_H_29_O_13_ | 243.0654(100%) | tetrahydroxystilbene-O-(arabinoyl)-hexoside ^a^ | 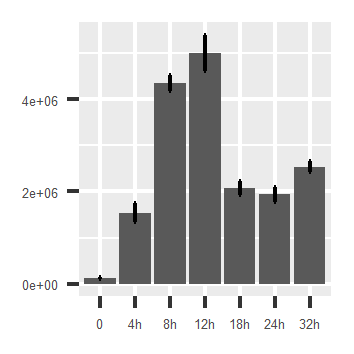 | | RRM, PRM 4h, 8h, 12h, 18h, 24, 32h |
| A33-2 | 12.27 | 537.1609 | 0.633 | C_25_H_29_O_13_ | 243.0653(100%) |  | 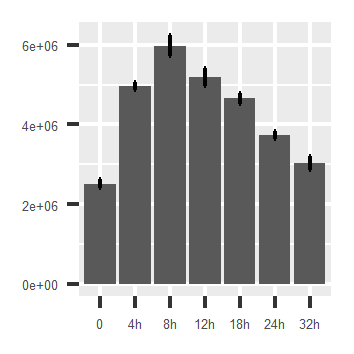 | | RRM, PRM 4h, 8h, 12h, 18h, 24, 32h |
| A34-1 | 16.9 | 541.1351 | -0.091 | C_27_H_25_O_12_ | 541.1337(20%) 405.1175(15%) 297.0610(70%) 243.0657(100%) 153.0179(45%) | tetrahydroxystilbene-O-hexoside-protocatechuic acid acyl (glycosyl hydroxyl moiety) ^a^ | 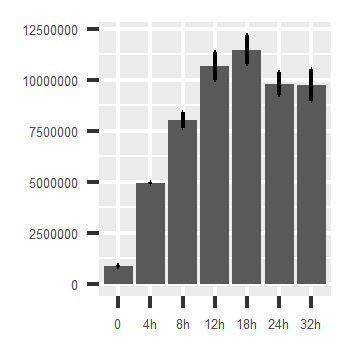 | | RRM, PRM 4h, 8h, 12h, 18h, 24, 32h |
| A34-2 | 19.16 | 541.1349 | -0.461 | C_27_H_25_O_12_ | 405.1172(15%) 297.0609(20%) 243.0655(100%) 153.0179(50%) |  |  | | PRM 4h, 8h, 12h, 18h, 24, 32h |
| A34-3 | 21.7 | 541.1352 | 0.094 | C_27_H_25_O_12_ | 379.1190(45%) 295.0957(35%) 243.0565(100%) | tetrahydroxystilbene-O-hexoside-protocatechuic acid acyl (phenolic hydroxyl moiety) ^a^ |  | | RRM, PRM 4h, 8h, 12h, 18h, 24, 32h |
| A34-4 | 22.07 | 541.1352 | 0.094 | C_27_H_25_O_12_ | 405.1170(5%) 379.0793(10%) 243.0568(100%) |  |  | | RRM, PRM 4h, 8h, 12h, 18h, 24, 32h |
| A35 | 12.92 | 543.1121 | 1.619 | C_26_H_23_O_13_ | 405.1196(15%) 243.0655(100%) | tetrahydroxystilbene-O- (furan-2, 5-dicarboxylic acid acyl)-hexoside ^b^ |  | | RRM, PRM 4h, 8h, 12h, 18h, 24, 32h |
| A36 | 19.1 | 543.1501 | -1.288 | C_27_H_27_O_12_ | 381.0962(10%) 337.1069(100%) 243.0657(45%) | tetrahydroxystilbene-O-hexoside-O-methoxymethyl-furancarboxylic acid acyl (phenolic hydroxyl moiety) ^b^ |  | | PRM 8h, 12h, 18h, 24, 32h |
| A37-1 | 11.86 | 547.1453 | -0.757 | C_26_H_27_O_13_ | 385.0914(100%) 243.0654(20%) | tetrahydroxystilbene-O-hexoside-oxoadipic acid acyl (phenolic hydroxyl moiety) ^b^ |  | | PRM 4h, 8h, 12h, 18h, 24, 32h |
| A37-2 | 12.15 | 547.1452 | -0.939 | C_26_H_27_O_13_ | 385.0913(100%) 243.0654(20%) |  |  | | PRM 4h, 8h, 12h, 18h, 24, 32h |
| A38-1 | 15.14 | 549.1611 | 0.833 | C_26_H_29_O_13_ | 405.1184(10%) 387.1066(10%) 243.0654(100%) | tetrahydroxystilbene-O-hexoside-hydroxyadipic acid acyl (phenolic hydroxyl moiety) ^b^ |  | | RRM, PRM 4h, 8h, 12h, 18h, 24, 32h |
| A38-2 | 16.86 | 549.1608 | 0.533 | C_26_H_29_O_13_ | 405.1184(10%) 387.1066(10%) 243.0654(100%) |  |  | | RRM, PRM 4h, 8h, 12h, 18h, 24, 32h |
| A38-3 | 17.35 | 549.1610 | 0.733 | C_26_H_29_O_13_ | 405.1184(10%) 387.1066(100%) 243.0654(10%) |  |  | | RRM, PRM 4h, 8h, 12h, 18h, 24, 32h |
| A39-1 | 20.64 | 551.1556 | -0.517 | C_29_H_27_O_11_ | 405.1180(10%) 243.0655(100%) 163.0386(10%) 145.0279(35%) | tetrahydroxystilbene-O-hexoside-*p*-hydroxycinnamoyl (phenolic hydroxyl moiety) ^a^ |  | | RRM, PRM 4h, 8h, 12h, 18h, 24, 32h |
| A39-2 | 20.79 | 551.1555 | -0.698 | C_29_H_27_O_11_ | 389.1017(100%) 243.0655(52%) 163.0386(10%) 145. 0279(20%) | tetrahydroxystilbene-O-(*p*-hydroxycinnamoyl)-hexoside ^a^ |  | | RRM, PRM 4h, 8h, 12h, 18h, 24, 32h |
| A39-3 | 21.85 | 551.1555 | -0.698 | C_29_H_27_O_11_ | 405.1180(10%) 307.0815(20%) 243.0655(100%) 163.0386(10%）145.0279(35%) |  |  | | RRM, PRM 4h, 8h, 12h, 18h, 24, 32h |
| A39-4 | 23.97 | 551.1557 | -0.335 | C_29_H_27_O_11_ | 405.1184(10%) 243.0657(100%) 163.0387(20%) 145.0280(40%) |  |  | | RRM, PRM 4h, 8h, 12h, 18h, 24, 32h |
| A39-5 | 24.11 | 551.1555 | -0.698 | C_29_H_27_O_11_ | 405.1178(10%) 243.0656(100%) 163.0387(20%) 145.0280(30%) |  |  | | RRM, PRM 4h, 8h, 12h, 18h, 24, 32h |
| A40-1 | 10.45 | 557.1293 | -1.371 | C_27_H_25_O_13_ | 405.1178(10%) 313.0555(40%) 243.0655(100%) 169.0127(20%) | tetrahydroxystilbene-O-hexoside-O-galloyl (glycosyl hydroxyl moiety) ^a^ |  | | RRM, PRM 4h, 8h, 12h, 18h, 24, 32h |
| A40-2 | 12.40 | 557.1291 | -1.73 | C_27_H_25_O_13_ | 405.1178(10%) 313.0555(60%) 243.0655(100%) 169.0127(40%) |  |  | | RRM, PRM 4h, 8h, 12h, 18h, 24, 32h |
| A40-3 | 13.16 | 557.1302 | 0.244 | C_27_H_25_O_13_ | 405.1178(10%) 313.0555(25%) 243.0655(100%) 169.0127(10%) |  |  | | RRM, PRM 4h, 8h, 12h, 18h, 24, 32h |
| A40-4 | 14.31 | 557.1294 | -1.192 | C_27_H_25_O_13_ | 405.1178(10%) 313.0555(20%) 243.0655(100%) 169.0127(30%) |  |  | | RRM, PRM 4h, 8h, 12h, 18h, 24, 32h |
| A40-5 | 14.68 | 557.1300 | -0.115 | C_27_H_25_O_13_ | 405.1178(5%) 313.0555(60%) 243.0655(70%) 169.0127(100%) |  |  | | RRM, PRM 4h, 8h, 12h, 18h, 24, 32h |
| A40-6 | 16.32 | 557.1301 | 0.065 | C_27_H_25_O_13_ | 405.1178(10%) 313.0555(70%) 243.0655(100%) 169.0127(45%) |  |  | | RRM, PRM 4h, 8h, 12h, 18h, 24, 32h |
| A40-7 | 16.71 | 557.1302 | 0.244 | C_27_H_25_O_13_ | 405.1178(10%) 313.0555(20%) 243.0655(100%) 169.0127(32%) |  |  | | RRM, PRM 4h, 8h, 12h, 18h, 24, 32h |
| A40-8 | 18.38 | 557.1295 | -1.012 | C_27_H_25_O_13_ | 405.1178(10%) 313.0555(10%) 243.0655(100%) 169.0127(8%) |  |  | | RRM, PRM 4h, 8h, 12h, 18h, 24, 32h |
| A41-1 | 14.79 | 561.1609 | -0.827 | C_27_H_29_O_13_ | 405.1174(20%) 243.0652(100%) | tetrahydroxystilbene-O- (gabosine C)-hexoside ^b^ |  | | PRM 18h, 24, 32h |
| A41-2 | 15.32 | 561.1609 | -0.827 | C_27_H_29_O_13_ | 405.1174(20%) 243.0652(100%) |  |  | | PRM 12h, 18h, 24, 32h |
| A42-1 | 5.65 | 567.1711 | -1.461 | C_26_H_31_O_14_ | 447.1278(30%)285.0761(100%)243.0662(25%) | tetrahydroxystilbene-O-  hexoside-C-glycoside ^a^ |  | | RRM, PRM 4h, 8h, 12h, 18h, 24, 32h |
| A42-5 | 8.94 | 567.1713 | -1.109 | C_26_H_31_O_14_ | 477.1378(25%)447.1298(35%)315.0864(78%)285.0765(100%)243.0661(28%) |  |  | | RRM, PRM 4h, 8h, 12h, 18h, 24, 32h |
| A42-6 | 9.14 | 567.1711 | -1.461 | C_26_H_31_O_14_ | 447.1280(20%)285.0764(100%)243.0662(10%) |  |  | | RRM, PRM 4h, 8h, 12h, 18h, 24, 32h |
| A42-2 | 6.65 | 567.1716 | -0.580 | C_26_H_31_O_14_ | 405.1164(15%)243.0655(100%) | tetrahydroxystilbene-O-di-hexosides ^a^ |  | | RRM, PRM 4h, 8h, 12h, 18h, 24, 32h |
| A42-3 | 7.91 | 567.1712 | -1.285 | C_26_H_31_O_14_ | 243.0655（100%） |  |  | | PRM 4h, 8h, 12h, 18h, 24, 32h |
| A42-4 | 8.53 | 567.1714 | -0.932 | C_26_H_31_O_14_ | 243.0652（100%） |  |  | | PRM 4h, 8h, 12h, 18h, 24, 32h |
| A42-7 | 10.02 | 567.1714 | -0.932 | C_26_H_31_O_14_ | 243.0652（100%） |  |  | | RRM, PRM 4h, 8h, 12h, 18h, 24, 32h |
| A42-8 | 10.21 | 567.1716 | -0.58 | C_26_H_31_O_14_ | 243.0654（100%） |  |  | | RRM, PRM 4h, 8h, 12h, 18h, 24, 32h |
| A42-9 | 10.43 | 567.1713 | -1.109 | C_26_H_31_O_14_ | 243.0655（100%） |  |  | | RRM, PRM 4h, 8h, 12h, 18h, 24, 32h |
| A42-10 | 10.88 | 567.1714 | -0.932 | C_26_H_31_O_14_ | 243.0652（100%） |  |  | | PRM 4h, 8h, 12h, 18h, 24, 32h |
| A42-11 | 12.09 | 567.1713 | -1.109 | C_26_H_31_O_14_ | 243.0654（100%） |  |  | | RRM, PRM 4h, 8h, 12h, 18h, 24, 32h |
| A42-12 | 13.54 | 567.1711 | -1.461 | C_26_H_31_O_14_ | 243.0655（100%） |  |  | | RRM, PRM 4h, 8h, 12h, 18h, 24, 32h |
| A42-13 | 14.05 | 567.1714 | -0.932 | C_26_H_31_O_14_ | 405.1154(10%)243.0653（100%） |  |  | | RRM, PRM 4h, 8h, 12h, 18h, 24, 32h |
| A42-14 | 18.29 | 567.1504 | -0.704 | C_29_H_27_O_12_ | 405.1180(13%)243.0655(100%)161.0230(52%) | tetrahydroxystilbene-O-(caffeoyl)-hexoside ^a^ |  | | RRM, PRM 4h, 8h, 12h, 18h, 24, 32h |
| A42-15 | 19.5 | 567.1504 | -0.704 | C_29_H_27_O_12_ | 323.0765(35%)243.0657(100%)161.0229(83%) |  |  | | RRM, PRM 4h, 8h, 12h, 18h, 24, 32h |
| A42-16 | 21.64 | 567.1501 | -1.233 | C_29_H_27_O_12_ | 243.0655(95%)161.0229(100%) |  |  | | RRM, PRM 4h, 8h, 12h, 18h, 24, 32h |
| A43-1 | 8.86 | 573.1245 | 0.618 | C_27_H_25_O_14_ | 243.0657(10%) 166.9971(90%)123.0071(100%) | tetrahydroxystilbene-O- (tetrahydroxybenzoic acid acyl)-hexosides ^b^ |  | | RRM, PRM 4h, 8h, 12h, 18h, 24, 32h |
| A43-2 | 10.22 | 573.1253 | 1.418 | C_27_H_25_O_14_ | 243.0657(10%) 166.9971(90%)123.0071(100%) |  |  | | PRM 4h, 8h, 12h, 18h, 24, 32h |
| A43-3 | 10.65 | 573.1246 | 0.718 | C_27_H_25_O_14_ | 243.0657(10%) 166.9971(90%)123.0071(100%) |  |  | | RRM, PRM 4h, 8h, 12h, 18h, 24, 32h |
| A43-4 | 10.87 | 573.1246 | 0.718 | C_27_H_25_O_14_ | 243.0657(10%) 166.9971(90%)123.0071(100%) |  |  | | RRM, PRM 4h, 8h, 12h, 18h, 24, 32h |
| A43-5 | 12.17 | 573.1251 | 1.218 | C_27_H_25_O_14_ | 243.0657(10%) 166.9971(90%)123.0071(100%) |  |  | | RRM, PRM 4h, 8h, 12h, 18h, 24, 32h |
| A43-6 | 12.66 | 573.1251 | 1.218 | C_27_H_25_O_14_ | 243.0657(10%) 166.9971(90%)123.0071(100%) |  |  | | RRM, PRM 4h, 8h, 12h, 18h, 24, 32h |
| A44 | 15.26 | 575.1402 | -0.745 | C_27_H_27_O_14_ | 337.0707(100%) 243.0648(20%) | tetrahydroxystilbene-O- (dioxoheptane-dicarboxylic acid acyl)-hexoside ^b^ |  | | PRM 12h, 18h, 24, 32h |
| A45-1 | 21.08 | 581.1655 | -1.634 | C_30_H_29_O_12_ | 405.1179(10%) 337.0921 (5%) 243.0655(100%) 193.0493(5%) 175.0387(20%) | tetrahydroxystilbene-O-hexoside-feruloyl (glycosyl hydroxyl moiety) ^a^ |  | | RRM, PRM 4h, 8h, 12h, 18h, 24, 32h |
| A45-2 | 22.49 | 581.1666 | 0.259 | C_30_H_29_O_12_ | 405.1163(5) 337.0921(13%) 243.0656(100%)193.0493(15%) 175.0387(25%) |  |  | | RRM, PRM 4h, 8h, 12h, 18h, 24, 32h |
| A45-3 | 23.74 | 581.1662 | -0.429 | C_30_H_29_O_12_ | 337.0917(20%) 243.0655(100%) 193.0493(5%) 175.0387(20%) |  |  | | PRM 4h, 8h, 12h, 18h, 24, 32h |
| A45-4 | 24.55 | 581.1661 | -0.601 | C_30_H_29_O_12_ | 405.1179(8%) 337.0921 (5%) 243.0655(100%) 193.0493(20%) 175.0387(30%) |  |  | | RRM, PRM 4h, 8h, 12h, 18h, 24, 32h |
| A45-5 | 24.74 | 581.1658 | -1.118 | C_30_H_29_O_12_ | 387.1073(8%) 337.0918 (5%) 243.0655(100%) 193.0493(20%) 175.0387(30%) |  |  | | RRM, PRM 4h, 8h, 12h, 18h, 24, 32h |
| A46 | 11.39 | 591.2075 | -1.377 | C_29_H_35_O_13_ | 405.1182(10%) 243.0654(100%) 185.0806(10%) | tetrahydroxystilbene-O- (hydroxynonanedioic acid acyl)-hexoside ^b^ |  | | PRM 4h, 8h, 12h, 18h, 24, 32h |
| A47 | 7.92 | 613.1769 | -0.828 | C_27_H_33_O_16_ | 405.1176(15%) 243.0655(100%) | tetrahydroxystilbene-O- (Glucoheptanoyl)-hexoside ^b^ |  | | RRM, PRM 4h, 8h, 12h, 18h, 24, 32h |
| A48-1 | 12.4 | 719.1826 | -0.399 | C_33_H_35_O_18_ | 557.1287(45%) 405.1174(10%) 313.0557(100%) 243.0655(60%) 169.0126(30%) | tetrahydroxystilbene-O-dihexoside-galloyl (glycosyl hydroxyl moiety) ^b^ |  | | RRM, PRM 4h, 8h, 12h, 18h, 24, 32h |
| A48-2 | 13.08 | 719.1829 | 0.018 | C_33_H_35_O_18_ | 557.1287(45%) 405.1174(10%) 313.0557(100%) 243.0655(60%) 169.0126(30%) |  |  | | RRM, PRM 4h, 8h, 12h, 18h, 24, 32h |
| A48-3 | 13.97 | 719.1825 | -0.538 | C_33_H_35_O_18_ | 557.1287(100%) 405.1170(25%) 313.0558(75%) 243.0653(68%) 169.0129(30%) |  |  | | PRM 4h, 8h, 12h, 18h, 24, 32h |
| A49-1 | 14.02 | 827.2400 | -0.486 | C_40_H_43_O_19_ | 421.1119(10%) 405.1174(30%) 259.0606(85%) 243.0653(100%) 165.0180(30%) | polygonumoside C/D ^a^ |  | | RRM, PRM 4h, 8h, 12h, 18h, 24, 32h |
| A49-2 | 14.31 | 827.2399 | -0.607 | C_40_H_43_O_19_ | 259.0607(100%) 243.0658(15%) |  |  | | RRM, PRM 4h, 8h, 12h, 18h, 24, 32h |
| A49-3 | 14.75 | 827.2395 | -1.090 | C_40_H_43_O_19_ | 405.1171(25%) 259.0607(50%) 243.0654(100%) 165.0178(40%) |  |  | | RRM, PRM 4h, 8h, 12h, 18h, 24, 32h |
| A49-4 | 15.03 | 827.2404 | -0.003 | C_40_H_43_O_19_ | 259.0607(100%) 243.0658(15%) |  |  | | RRM, PRM 4h, 8h, 12h, 18h, 24, 32h |
| A49-5 | 15.51 | 827.2401 | -0.365 | C_40_H_43_O_19_ | 405.1171(20%) 259.0607(30%) 243.0654(100%) 165.0178(40%) |  |  | | RRM, PRM 4h, 8h, 12h, 18h, 24, 32h |
| A49-6 | 15.66 | 827.2404 | -0.003 | C_40_H_43_O_19_ | 405.1171(20%) 259.0607(90%) 243.0654(100%) 165.0178(40%) |  |  | | RRM, PRM 4h, 8h, 12h, 18h, 24, 32h |
| A50-1 | 19.88 | 837.2604 | -0.881 | C_42_H_45_O_18_ | 675.2068(13%) 513.1556(38%) 431.1342(35%)405.1165(10%)269.0816(100%) 243.0659(70%) | polygonumnolide D ^a^ |  | | RRM, PRM 4h, 8h, 12h, 18h, 24, 32h |
| A50-2 | 20.49 | 837.2601 | -1.239 | C_42_H_45_O_18_ | 675.2068(13%) 513.1541(45%) 431.1342(30%)405.1165(10%)269.0816(100%) 243.0659(68%) |  |  | | RRM, PRM 4h, 8h, 12h, 18h, 24, 32h |
| A50-3 | 22.33 | 837.2606 | -0.642 | C_42_H_45_O_18_ | 675.2068(13%) 513.1541(38%) 431.1342(25%)405.1165(10%)269.0816(100%) 243.0659(60%) |  |  | | RRM, PRM 4h, 8h, 12h, 18h, 24, 32h |
| A50-4 | 22.81 | 837.2610 | -0.164 | C_42_H_45_O_18_ | 675.2068(13%) 513.1541(38%) 431.1342(30%)405.1165(20%)269.0816(100%) 243.0659(60%) |  |  | | RRM, PRM 4h, 8h, 12h, 18h, 24, 32h |
| A50-5 | 23.37 | 837.2603 | -1.000 | C_42_H_45_O_18_ | 431.1342(30%)405.1165(10%)269.0816(100%) 243.0659(60%) |  |  | | RRM, PRM 4h, 8h, 12h, 18h, 24, 32h |
| A50-6 | 23.66 | 837.2606 | -0.642 | C_42_H_45_O_18_ | 431.1342(10%)405.1165(30%)269.0816(85%) 243.0659(100%) |  |  | | PRM 4h, 8h, 12h, 18h, 24, 32h |
| A50-7 | 23.77 | 837.2606 | -0.642 | C_42_H_45_O_18_ | 675.2068(13%) 513.1541(38%) 431.1342(10%)405.1165(35%)269.0816(75%) 243.0659(100%) |  |  | | PRM 4h, 8h, 12h, 18h, 24, 32h |
| A50-8 | 25.78 | 837.2607 | -0.523 | C_42_H_45_O_18_ | 431.1337(48%)405.1165(30%)269.0816(100%) 243.0659(85%) |  |  | | RRM, PRM 4h, 8h, 12h, 18h, 24, 32h |
| A51-1 | 16.46 | 841.2553 | -0.894 | C_41_H_45_O_19_ | 405.1182(20%) 273.0764(40%) 243.0654(100%） | methylation polygonumoside C/D ^b^ |  | | RRM, PRM 4h, 8h, 12h, 18h, 24, 32h |
| A51-2 | 16.61 | 841.2553 | -0.894 | C_41_H_45_O_19_ | 405.1180(20%) 273.0764(40%) 243.0654(100%） |  |  | | RRM, PRM 4h, 8h, 12h, 18h, 24, 32h |
| A51-3 | 18.13 | 841.2556 | -0.537 | C_41_H_45_O_19_ | 405.1180(20%) 273.0764(25%) 243.0654(100%） |  |  | | RRM, PRM 4h, 8h, 12h, 18h, 24, 32h |
| A51-4 | 18.62 | 841.2552 | -1.013 | C_41_H_45_O_19_ | 405.1180(20%) 273.0764(25%) 243.0654(100%） |  |  | | RRM, PRM 4h, 8h, 12h, 18h, 24, 32h |
| A51-5 | 18.8 | 841.2551 | -1.132 | C_41_H_45_O_19_ | 405.1177(20%) 273.0764(25%) 243.0654(100%） |  |  | | RRM, PRM 4h, 8h, 12h, 18h, 24, 32h |
| A52 | 19.14 | 853.2560 | -0.061 | C_42_H_45_O_19_ | 447.1300(30%) 405.1175(25%) 285.0765(100%) 243.0656(50%) | hydroxylation polygonumnolide D ^b^ |  | | PRM 4h, 8h, 12h, 18h, 24, 32h |
| A53-1 | 16.09 | 857.2507 | -0.311 | C_41_H_45_O_20_ | 405.1172(10%) 289.0709(10%) 257.0448(100%) 243.0654(50%) | hydroxylation methylation polygonumoside C/D ^b^ |  | | RRM, PRM 4h, 8h, 12h, 18h, 24, 32h |
| A53-2 | 16.21 | 857.2503 | -0.778 | C_41_H_45_O_20_ | 405.1172(25%) 289.0709(10%) 257.0448(100%) 243.0654(95%) |  |  | | PRM 4h, 8h, 12h, 18h, 24, 32h |
| A53-3 | 16.63 | 857.2502 | -0.894 | C_41_H_45_O_20_ | 405.1172(10%) 289.0709(10%) 257.0448(100%) 243.0654(50%) |  |  | | RRM, PRM 4h, 8h, 12h, 18h, 24, 32h |
| A53-4 | 17.21 | 857.2504 | -0.661 | C_41_H_45_O_20_ | 405.1172(10%) 289.0709(10%) 257.0448(100%) 243.0654(50%) |  |  | | RRM, PRM 4h, 8h, 12h, 18h, 24, 32h |
| B1-1 | 7.99 | 389.1240 | -0.490 | C_20_H_21_O_8_ | 227.0702 | isomer polydatin ^a^ |  | | RRM, PRM 4h, 8h, 12h, 18h, 24, 32h |
| B1-2 | 10.31 | 389.1243 | 0.281 | C_20_H_21_O_8_ | 227.0705 | polydatin ^C^ |  | | RRM, PRM 4h, 8h, 12h, 18h, 24, 32h |
| B1-3 | 14.5 | 389.1242 | 0.024 | C_20_H_21_O_8_ | 227.0703 | isomer polydatin ^a^ |  | | RRM, PRM 4h, 8h, 12h, 18h, 24, 32h |
| B2-1 | 14.41 | 541.1345 | -1.200 | C_27_H_25_O_12_ | 541.1346(80%)313.0559(100%)227.0702(45%) 169.0128(70%) | trihydroxystilbene-O-hexoside-O-galloyl (glycosyl hydroxyl moiety) ^a^ |  | | RRM, PRM 4h, 8h, 12h, 18h, 24, 32h |
| B2-2 | 15.26 | 541.1344 | -1.385 | C_27_H_25_O_12_ | 313.0557(100%)227.0702(20%)169.0128(35%) |  |  | | RRM, PRM 4h, 8h, 12h, 18h, 24, 32h |
| B2-3 | 15.98 | 541.1348 | -0.646 | C_27_H_25_O_12_ | 541.1342(50%)313.0559(60%)227.0702(40%) 169.0127(100%) |  |  | | RRM, PRM 4h, 8h, 12h, 18h, 24, 32h |
| B2-4 | 16.13 | 541.1345 | -1.200 | C_27_H_25_O_12_ | 541.1342(40%)313.0559(60%)227.0702(30%) 169.0127(100%) |  |  | | RRM, PRM 4h, 8h, 12h, 18h, 24, 32h |
| B3-1 | 16.47 | 457.1136 | -0.919 | C_23_H_21_O_10_ | 295.0605(100%) 227.0702(20%) | trihydroxystilbene-O-hexoside-O- acid deltique acyl (phenolic hydroxyl moiety) ^b^ |  | | RRM, PRM 4h, 8h, 12h, 18h, 24, 32h |
| B3-2 | 17.83 | 457.1136 | -0.919 | C_23_H_21_O_10_ | 295.0605(100%) 227.0702(20%) |  |  | | RRM, PRM 4h, 8h, 12h, 18h, 24, 32h |
| B4 | 12.55 | 535.1816 | -0.933 | C_26_H_31_O_12_ | 227.0702(100%) | trihydroxystilbene-(deoxyhexose)-O-hexoside ^a^ |  | | PRM 4h, 8h, 12h, 18h, 24, 32h |
| B5 | 13.17 | 359.1132 | -1.187 | C_19_H_19_O_7_ | 359.1129(95%) 227.0701(100%) | trihydroxystilbene-O-pentose ^a^ |  | | PRM 4h, 8h, 12h, 18h, 24, 32h |
| C1-1 | 2.42 | 421.1138 | -0.522 | C_20_H_21_O_10_ | 259.0609(100%) | pentahydroxystilbene glycoside ^a^ |  | | RRM, PRM 4h, 8h, 12h, 18h, 24, 32h |
| C1-2 | 2.8 | 421.1136 | -0.997 | C_20_H_21_O_10_ | 259.0609(100%) |  |  | | RRM, PRM 4h, 8h, 12h, 18h, 24, 32h |
| C1-3 | 3.04 | 421.1136 | -0.997 | C_20_H_21_O_10_ | 259.0609(100%) |  |  | | RRM, PRM 4h, 8h, 12h, 18h, 24, 32h |
| C1-4 | 3.81 | 421.1137 | -0.760 | C_20_H_21_O_10_ | 259.0609(100%) |  |  | | RRM, PRM 4h, 8h, 12h, 18h, 24, 32h |
| C1-5 | 4.54 | 421.1138 | -0.522 | C_20_H_21_O_10_ | 259.0609(100%) |  |  | | RRM, PRM 4h, 8h, 12h, 18h, 24, 32h |
| C1-6 | 7.55 | 421.1136 | -0.997 | C_20_H_21_O_10_ | 259.0609(100%) |  |  | | RRM, PRM 4h, 8h, 12h, 18h, 24, 32h |
| C1-7 | 9.12 | 421.1140 | -0.048 | C_20_H_21_O_10_ | 259.0609(100%) |  |  | | RRM, PRM 4h, 8h, 12h, 18h, 24, 32h |
| C2 | 14.05 | 545.1291 | -1.768 | C_26_H_25_O_13_ | 421.1128(15%) 259.0609(100%) 123.0070(25%) | pentahydroxystilbene -(5-HMF)-O-hexoside ^b^ |  | | RRM, PRM 4h, 8h, 12h, 18h, 24, 32h |
| D1-1 | 10.15 | 403.1030 | -1.130 | C_20_H_19_O_9_ | 241.0497(100%) | tetrahydroxy-phenanthrene-O-hexoside ^a^ |  | | RRM, PRM 4h, 8h, 12h, 18h, 24, 32h |
| D1-2 | 12.22 | 403.1030 | -1.130 | C_20_H_19_O_9_ | 241.0496(100%) |  |  | | RRM, PRM 4h, 8h, 12h, 18h, 24, 32h |
| D2-1 | 3.86 | 549.1605 | -1.573 | C_26_H_29_O_13_ | 387.1072(55%)297.0760(100%)281.0450(42%) 241.0497(38%) | tetrahydroxy-phenanthrene-O-hexoside-O-*p*-hydroxycinnamoyl (phenolic hydroxyl moiety) ^a^ |  | | PRM 4h, 8h, 12h, 18h, 24, 32h |
| D2-2 | 5.48 | 549.1611 | -0.481 | C_26_H_29_O_13_ | 387.1072(55%)297.0760(100%)281.0450(42%) 241.0497(38%) |  |  | | RRM, PRM 4h, 8h, 12h, 18h, 24, 32h |
| D2-3 | 7.01 | 549.1610 | -0.663 | C_26_H_29_O_13_ | 387.1072(55%)297.0760(100%)281.0450(42%) 241.0497(38%) |  |  | | RRM, PRM 4h, 8h, 12h, 18h, 24, 32h |
| D2-4 | 7.85 | 549.1605 | -1.573 | C_26_H_29_O_13_ | 387.1072(55%)297.0760(100%)281.0450(42%) 241.0497(38%) |  |  | | PRM 4h, 8h, 12h, 18h, 24, 32h |
| D2-5 | 8.72 | 549.1608 | -1.027 | C_26_H_29_O_13_ | 387.1072(55%)297.0760(100%)281.0450(42%) 241.0497(38%) |  |  | | PRM 4h, 8h, 12h, 18h, 24, 32h |
| D2-6 | 9.59 | 549.1608 | -1.027 | C_26_H_29_O_13_ | 387.1072(55%)297.0760(100%)281.0450(42%) 241.0497(38%) |  |  | | PRM 4h, 8h, 12h, 18h, 24, 32h |
| D2-7 | 10.01 | 549.1609 | -0.845 | C_26_H_29_O_13_ | 387.1072(55%)297.0760(100%)281.0450(42%) 241.0497(38%) |  |  | | PRM 4h, 8h, 12h, 18h, 24, 32h |
| D2-8 | 11.58 | 549.1605 | -1.573 | C_26_H_29_O_13_ | 387.1072(55%)297.0760(100%)281.0450(42%) 241.0497(38%) |  |  | | PRM 4h, 8h, 12h, 18h, 24, 32h |
| E1 | 8.99 | 407.1343 | -1.119 | C_20_H_23_O_9_ | 245.0811(100%) | dihydrotetrahydroxystilbene-O-hexoside ^a^ |  | | RRM, PRM 4h, 8h, 12h, 18h, 24, 32h |
| E2-1 | 20.45 | 527.1552 | -1.299 | C_27_H_27_O_11_ | 365.1017(100%) 335.0918(65%) 245.0814(25%) | dihydrotetrahydroxystilbene-O-hexoside-salicylic acid acyl (phenolic hydroxyl moiety) ^a^ |  | | PRM 8h, 12h, 18h, 24, 32h |
| E2-2 | 20.78 | 527.1546 | -2.437 | C_27_H_27_O_11_ | 365.1017(100%) 335.0918(65%) 245.0814(25%) |  |  | | RRM, PRM 4h, 8h, 12h, 18h, 24, 32h |
| E3 | 19.21 | 539.1766 | -0.768 | C_25_H_31_O_13_ | 245.0811(100%) | dihydrotetrahydroxystilbene-O-(pentose)-hexoside ^a^ |  | | RRM, PRM 4h, 8h, 12h, 18h, 24, 32h |
| F1-1 | 9.67 | 419.0980 | -0.883 | C_20_H_19_O_10_ | 257.0452(100%) | pentahydroxy-phenanthrene-O-hexoside ^a^ |  | | RRM, PRM 4h, 8h, 12h, 18h, 24, 32h |
| F1-2 | 10.28 | 419.0980 | -0.883 | C_20_H_19_O_10_ | 257.0452(100%) |  |  | | RRM, PRM 4h, 8h, 12h, 18h, 24, 32h |
| G1 | 20.48 | 373.1286 | 0.421 | C_20_H_21_O_7_ | 211.0751(100%) | dihydroxystilbene-O-hexoside ^a^ |  | | PRM 4h, 8h, 12h, 18h, 24, 32h |
| G2-1 | 23.92 | 525.1396 | 0.462 | C_27_H_25_O_11_ | 525.1392(50%) 313.0558(55%) 211.0753(30%) 169.0128(100%) 151.0020(20%) | dihydroxystilbene-O-hexoside--O-galloyl (glycosyl hydroxyl moiety) ^a^ |  | | RRM, PRM 4h, 8h, 12h, 18h, 24, 32h |
| G2-2 | 25.81 | 525.1394 | 0.262 | C_27_H_25_O_11_ | 525.1392(50%) 313.0558(55%) 211.0753(30%) 169.0128(100%) 151.0020(20%) |  |  | | PRM 4h, 8h, 12h, 18h, 24, 32h |

^a^ It had been reported in *Polygonum*

^b^ The potential new compounds.

^c^ compared with standard compounds.
