# Supplementary figures and images for: Transformation of Stilbene Glucosides From Reynoutria multiflora During Processing
Source: Front Pharmacol. 2022 Apr 25;13:757490. doi: 10.3389/fphar.2022.757490 (PMC9082504; doi:10.3389/fphar.2022.757490)

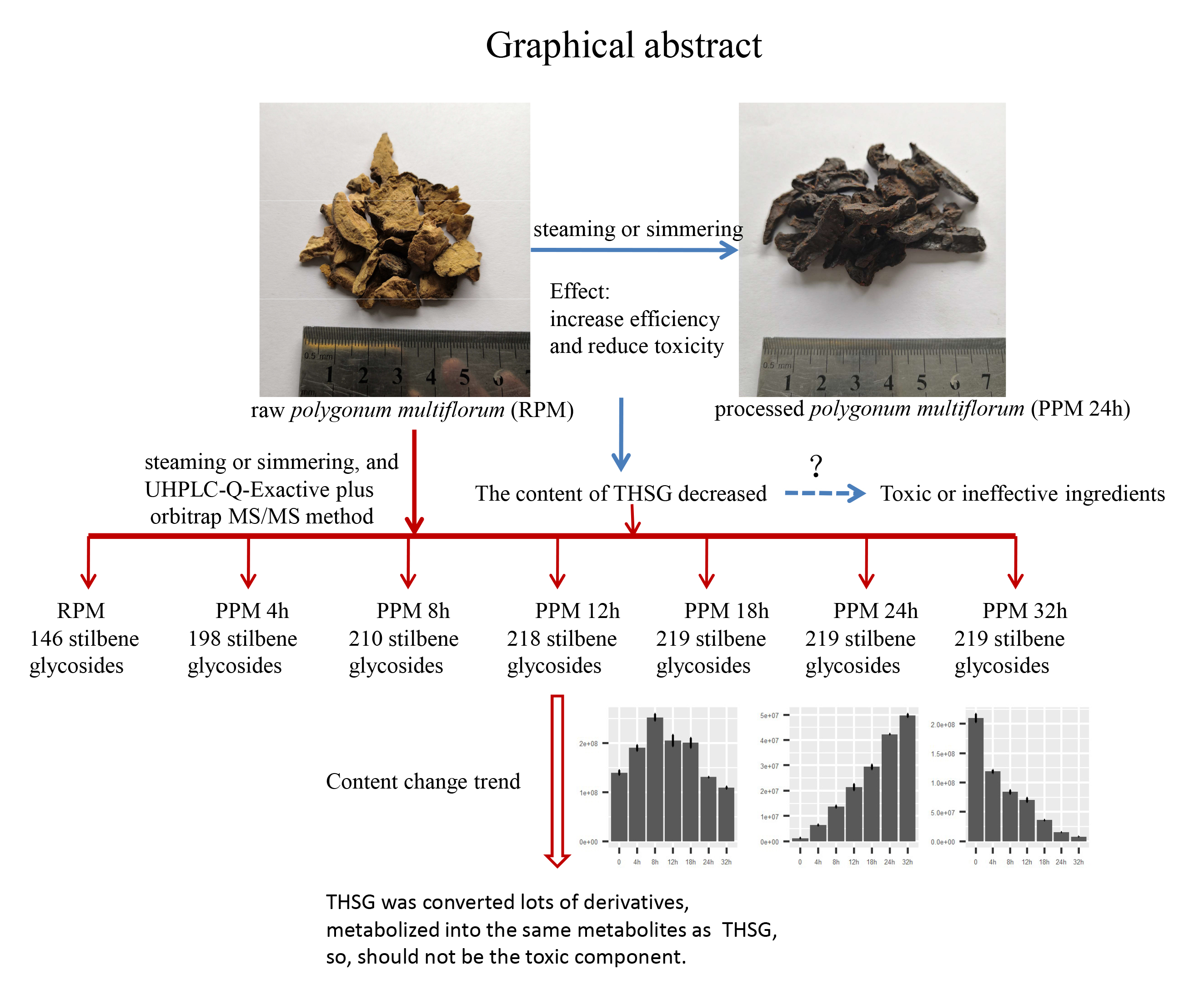

Supplement: Supplementary file 2 [file Image1.JPEG]
